# Differential Expression of Anthocyanin Biosynthetic Genes in Relation to Anthocyanin Accumulation in the Pericarp of *Litchi Chinensis* Sonn

**DOI:** 10.1371/journal.pone.0019455

**Published:** 2011-04-29

**Authors:** Yong-Zan Wei, Fu-Chu Hu, Gui-Bing Hu, Xiao-Jing Li, Xu-Ming Huang, Hui-Cong Wang

**Affiliations:** 1 Physiological Laboratory for South China Fruits, College of Horticulture, South China Agricultural University, Guangzhou, China; 2 The South Subtropical Crops Research Institute, Chinese Academy of Tropical Agricultural Sciences, Zhanjiang, Guangdong, China; 3 Institute of Tropical Fruit Trees, Hainan Academy of Agricultural Sciences, Haikou, China; United States Department of Agriculture, Agricultural Research Service, United States of America

## Abstract

Litchi has diverse fruit color phenotypes, yet no research reflects the biochemical background of this diversity. In this study, we evaluated 12 litchi cultivars for chromatic parameters and pigments, and investigated the effects of abscisic acid, forchlorofenron (CPPU), bagging and debagging treatments on fruit coloration in cv. Feizixiao, an unevenly red cultivar. Six genes encoding chalcone synthase (CHS), chalcone isomerase (CHI), flavanone 3-hydroxylase (F3H), dihydroflavonol 4-reductase (DFR), anthocyanidin synthase (ANS) and UDP-glucose: flavonoid 3-*O*-glucosyltransferase (UFGT) were isolated from the pericarp of the fully red litchi cv. Nuomici, and their expression was analyzed in different cultivars and under the above mentioned treatments. Pericarp anthocyanin concentration varied from none to 734 mg m^−2^ among the 12 litchi cultivars, which were divided into three coloration types, i.e. non-red (‘Kuixingqingpitian’, ‘Xingqiumili’, ‘Yamulong’and ‘Yongxing No. 2′), unevenly red (‘Feizixiao’ and ‘Sanyuehong’) and fully red (‘Meiguili’, ‘Baila’, Baitangying’ ’Guiwei’, ‘Nuomici’ and ‘Guinuo’). The fully red type cultivars had different levels of anthocyanin but with the same composition. The expression of the six genes, especially *LcF3H*, *LcDFR*, *LcANS* and *LcUFGT*, in the pericarp of non-red cultivars was much weaker as compared to those red cultivars. Their expression, *LcDFR* and *LcUFGT* in particular, was positively correlated with anthocyanin concentrations in the pericarp. These results suggest the late genes in the anthocyanin biosynthetic pathway were coordinately expressed during red coloration of litchi fruits. Low expression of these genes resulted in absence or extremely low anthocyanin accumulation in non-red cultivars. Zero-red pericarp from either immature or CPPU treated fruits appeared to be lacking in anthocyanins due to the absence of UFGT expression. Among these six genes, only the expression of UFGT was found significantly correlated with the pericarp anthocyanin concentration (r = 0.84). These results suggest that UFGT played a predominant role in the anthocyanin accumulation in litchi as well as pericarp coloration of a given cultivar.

## Introduction

Pigmentation is an appealing feature of fruits. Among the four pigment groups, i.e. anthocyanins, betalains, chlorophylls and carotenoids, anthocyanins are the most prominent imparting red, blue and black hues to the fruits in which they accumulate [1].

Anthocyanin biosynthesis is probably the most thoroughly studied plant secondary metabolism pathway. The metabolic pathway leading to their production has been well characterised in some model plants [2]. This pathway is usually divided into two sections, the early and the late sections [3]. The early sections leads to the formation of the dihydro-flavonols, comprising phenylalanine ammonialyase (PAL), cinnimate 4-hydroxylase (C4H), 4-coumarate: CoA ligase (4CL), chalcone synthase (CHS), chalcone isomerase (CHI), and flavanone 3-hydroxylase (F3H). Genes of these enzymes in the early section are here called the early genes. The late section leads to the formation of the anthocyanin molecule involving actions of dihydroflavonol reductase (DFR), anthocyanidin synthase (ANS) and UDPGlucose: flavonoid 3-*O*-glucosyltranferase (UFGT). Genes expressing the three enzymes are thus called the late genes in anthocyanin biosynthesis.

Litchi (*Litchi chinensis* Sonn.) is one of the important subtropical fruit crops, which is indigenous to South China. Red color on litchi fruit is the expression of anthocyanins [4], [5], [6]. Anthocyanin-accumulating fruit often display a range of intermediary colors from green to pink, then red or blue and finally purple to black with increasing anthocyanin and decreasing chlorophyll levels [7]. Litchi has diverse varieties with different fruit colors, yet no research reflects the biochemical background of this diversity. The diversity of fruit coloration in litchi genotypes provides interesting experimental materials for litchi anthocyanin studies.

Cloning of the structural genes in the anthocyanin biosynthetic pathway and the identification of genes encoding transcription factors that regulate the expression of the structural genes have been extensively reported in fruit crops because of market acceptance and health benefits. The expression of the UDP-glucose: flavonoid 3-*O*-glucosyltransferase (UFGT) gene was critical for anthocyanin biosynthesis in the grape berry [8]. White grape cultivars appear to be lacking in anthocyanins because of the absence of UFGT [9]. In apple fruits, five anthocyanin biosynthetic genes, *CHS*, *F3H*, *DFR*, *ANS* and *UFGT*, are coordinately expressed during red coloration in skin and their levels of expression are positively related to anthocyanin concentration [10]. Recently, studies indicate that expression of biosynthetic genes in anthocyanin accumulation is regulated by MYB transcription factor in the fruit of grapes [11], apples [12], [13], mangosteen [14], Chinese bayberries [15] and red pear [16].

In litchi, however, the information on molecular physiology of anthocyanin biosynthesis is quite limited. More data are available concerning anthocyanin concentration and composition changes during fruit development [4], [5] and coloration improved by bagging or spraying growth regulators [17], [18]. In this study, we cloned six structural genes of anthocyanin biosynthetic enzymes, CHS, CHI, F3H, DFR, ANS and UFGT and studied the expression of these genes in cultivars of three different color types. Effects of abscisic acid (ABA), forchlorofenron (CPPU) and cluster bagging and debagging treatments on anthocyanin accumulation and the expression of the genes in the pericarp were also examined.

## Results

### Pericarp color

**Figure 1:**
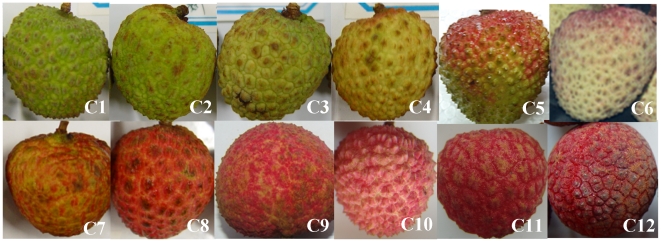
Appearance of 12 litchi cultivars. C1, ‘Kuixingqingpitian’; C2, ‘Xinqiumili’; C3, ‘Yamulong’; C4, ‘Yongxing No. 2′; C5, ‘Feizixiao’; C6, ‘Sanyuehong’; C7, ‘Meiguili’; C8, ‘Baila’; C9, ‘Baitangying’; C10, ‘Guiwei’; C11, ‘Nuomici’; C12, ‘Guinuo’.

The differences in pericarp color among the cultivars tested, expressed as the Hunter L*, a*, b*, and hue angle (h*) are shown in Table 1. Different cultivars displayed significant differences in color parameters. Basically, Hunter L*, b* showed a gradual decrease, while Hunter a* gradually increased as fruit color changed from green to light green-yellow, to yellow-red and to dark red among the cultivars (as shown in Figure 1). Hue angle (h*) derived from Hunter a* and b* color space, and therefore is a more practical parameter in reflecting fruit color. The h* value of ‘Kuixingqingpitian’, ‘Xingqiumili’, ‘Yamulong’ and ‘Yongxing No. 2′ were always significantly higher than those of ‘Feizixiao’, ‘Sanyuehong’, ‘Meiguili’, ‘Baila’, ‘Baitangying’, ‘Guiwei’, ‘Nuomici’ and ‘Guinuo’. The lower the hue angle, the redder the fruit skin. This result was consistent with the visual fruit color phenotypes.

**Table 1:** Color parameters (L*, a*, b*) and hue angle (h*) of litchis at maturity.

Cultivars	L*	a*	b*	h*
‘Kuixingqingpitian’	42.9±0.40de	−11.0±0.45h	30.8±0.46bc	109.5±0.59a
‘Xingquimili’	50.3±0.76a	−7.9±0.86gh	40.0±0.66a	107.1±5.52a
‘Yamulong’	44.7±0.65cd	−5.4±0.35g	32.6±0.46b	99.3±0.56b
‘Yongxing No. 2’	46.7±0.63bc	5.7±1.31f	33.1±0.72b	80.0±2.36c
‘Feizixiao’	39.9±0.83f	8.4±1.47ef	26.2±1.45d	72.0±3.06d
‘Sanyuehong’	47.8±1.25b	9.4±2.60e	29.6±1.59c	70.4±4.93d
‘Meiguili’	43.4±0.97de	13.6±1.03d	29.7±0.87c	65.1±2.13d
‘Baila’	41.8±1.21ef	20.4±1.65c	26.7±0.71d	53.3±2.74e
‘Baitangying’	36.8±0.66g	22.8±1.17bc	22.3±0.49e	45.0±1.88f
‘Guiwei’	41.6±0.60ef	25.2±0.67ab	22.0±0.53e	41.2±1.24f
‘Nuomici’	37.1±0.99g	22.2±0.55bc	18.01±0.59f	39.0±1.39f
‘Guinuo’	31.9±0.31h	27.0±0.27a	15.00±0.16g	29.1±0.40g

For each cultivar, means within a column followed by different letters are significantly different at p<0.05. Results of ANOVA test (n = 15) are presented in Table S1.

### Concentration of anthocyanins, chlorophylls and carotenoids and their correlations with hue angle

Anthocyanins, chlorophylls and carotenoids are almost exclusively found in the pericarp of litchi but not equally distributed within the pericarp. Anthocyanins and chlorophylls present mainly in the outer cell layers of the pericarp [19]. Therefore, concentration of the pigments on per square meter basis will be more applicable than on per gram basis to the comparison among different cultivars.

**Figure 2:**
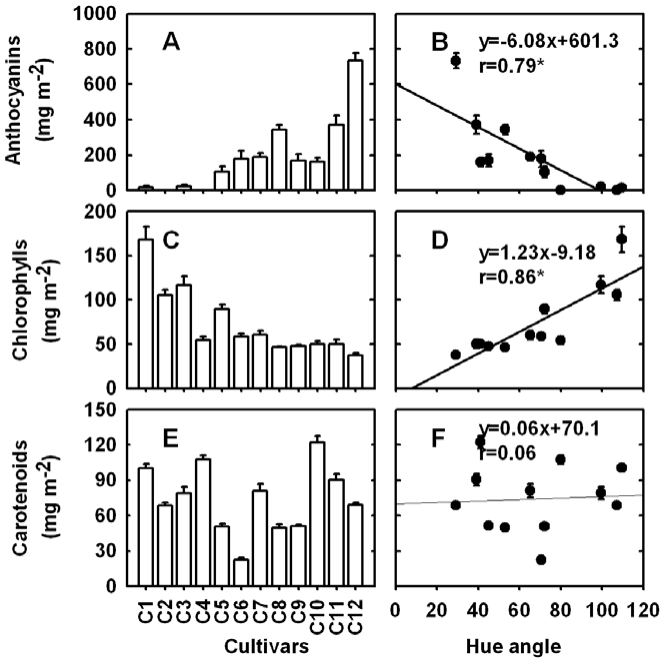
Concentrations of anthocyanins (A), chlorophylls (C), and carotenoids (E) and their correlations with hue angle (B, D, F) in the pericarp of twelve litchi cultivars. Each point is mean ± standard error (n = 15). C1 to C12 are different cultivars explained in Figure 1. Relative coefficient r with ‘*’ indicated significantly correlated at the level of P<0.05.

Total anthocyanin concentration was measured using the pH-differential spectrum method. Fruit color was mainly influenced by the concentration and distribution of anthocyanins in the skin. Anthocyanin concentration in the 12 cultivars ranged from none to 734 mg m^−2^ (Fig. 2A). ‘Kuixingqingpitian’, ‘Xingqiumili’, ‘Yamulong’ and ‘Yongxing No. 2′ contained extremely low or non-detectable levels of anthocyanins, while the rest cultivars accumulated quite a bit anthocyanins. In our study, anthocyanin levels of the tested cultivars were significantly negatively correlated with their hue angles (r = −0.78) (Fig. 2 B), which is consistent with sweet cherry [20]. Contrarily, the concentrations of chlorophylls in the pericarp of ‘Kuixingqingpitian’, ‘Xingqiumili’, and ‘Yamulong’ were much higher than those in the rest cultivars (Fig. 2 C). And the concentrations of chlorophylls were significantly positively correlated with their hue angles (r = 0.86) (Fig. 2 D). The pericarp of litchi contained carotenoids at levels ranging from 22 mg m^−2^ in cultivar ‘Sanyuehong’ to 122 mg m^−2^ in ‘Guiwei’ but displayed no visible patterns among the tested cultivars (Fig. 2 E, F).

According to the color appearance and concentrations and distribution of anthocyanins and chlorophylls, the tested 12 cultivars could be basically divided into three types: (a) the non-red ones that accumulate no or extremely low anthocyanins, including ‘Quixingqingpitian’, ‘Xingqiumili’, ‘Yamulong’ and ‘Yongxing No. 2′; (b) the unevenly red cultivars, ‘Feizixiao’ and ‘Sanyuehong’, which accumulate some anthocyanins while retaining relatively high levels of chlorophylls; (c) the evenly red cultivars that accumulate significant amount of anthocyanins with decreased chlorophylls, including ‘Meiguili’, ‘Baila’, ‘Baitangying’, ‘Guiwei’, ‘Nuomici’ and ‘Guinuo’ which display a serial color progressing from pink to dark red.

### Composition and relative content of anthocyanins in the pericarp of red litchi

**Figure 3:**
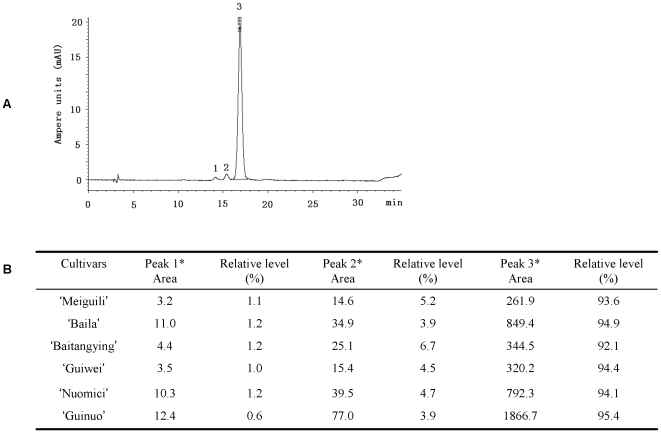
HPLC elution profile and peak area of individual anthocyanins. All the red cultivars examined contained the same three peaks: A) typical HPLC elution profile of anthocyanins from pericarp of litchi cv. Nuomici; B) anthocyanin compositions and their relative levels in the pericarp of red litchi cultivars. Asterisk represents that peak 1 to 3 were cyanidin (Peak 1), cyanidin-3-glucoside (Peak 2), cyanidin-3-rutinoside (Peak 3) respectively, which were putatively identified through the comparison of retention time and spectrum characters with the published data (Lee and Wicker,1991; Rivera-López et al,1999; Zhang et al., 2004). HPLC elution profiles of anthocyanins from pericarp of the rest cultivars are presented in Figure S1.

Previous works using reverse-phase high performance liquid chromatography (HPLC) revealed that the major pigment in ‘Brewster’ was cyanidin-3-rutinoside, and the minor pigments indentified were cyanidin-3-glucoside and malvidin-3-acetylglucoside [4], [5]. Zhang et al confirmed that the major anthocyanin in ‘Huaizhi’ was cyanidin-3-rutinoside (>91%) using HPLC equipped with mass spectrometry [6]. However, there is no available information by HPLC on other red litchi cultivars. To examine the composition and relative content of individual anthocyanins in red litchi varieties, anthocyanins were extracted and analyzed by HPLC. A very similar pattern of HPLC elution profiles for all the six red varieties was obtained. An example of a typical elution profile of red cultivar ‘Nuomici’ is shown in Fig. 3 A.

All the red cultivars examined contained the same three peaks and displayed similar relative levels (Fig 3 B). Peak 3 was the dominant anthoycanin in litchi pericarp, which was putatively identified as cyanidin-3-rutinoside through the comparison of retention time and spectrum characters with the published data [4], [5], [6]. The relative peak area of this compound (Peak 3) was around 94% in the six red cultivars. Peak 1 and Peak 2 which putatively identified as cyanidin and cyanidin-3-glucoside represented less abundant anthocyanins which had a relative area around 1% and 5%, respectively. These results indicated that red litchi cultivars had the same composition of anthocyanins and displayed similar relative levels of these three anthocyanins.

### Isolation and sequence analysis of anthocyanin biosynthetic genes

Fragments of the anthocyanin biosynthetic genes were isolated following the traditional cloning procedures including RT-PCR and TA ligation from ‘Nuomici’. Six anthocyanin biosynthetic genes, including *LcCHS* (450 bp), *LcCHI* (300 bp), *LcF3H* (450 bp), *LcDFR* (250 bp), *LcANS* (430 bp) and *LcUFGT* (950 bp), were obtained using degenerate primers (Table 2).

**Table 2:** Degenerate primers for cloning of anthocyanin biosynthetic genes in litchi pericarp.

Gene	Forward primer (5′ to 3′)	Reverse primer (5′ to 3′)	Product (bp)
*CHS*	GAGAAGTTCAAGCGCATGTGTGA	CCACGGAAAGTGACTGCAGTGAT	450
*CHI*	TTCCTCGGCGGCGCAGGGGWGAG	CTTCTGCATCAGTGTAAATTCC	300
*F3H*	TGGCGTGAAATWGTGACCTAYTT	TTCTTGAACCTCCCATTGCTCA	450
*DFR*	GAATCCAAGGATCCYGAGAAYGA	AAGTACATCCATCCAGTCATYTT	250
*ANS*	AAGGAGAAGTATGCMAATGAYC	AARAGCTGCAGRCCVGGRACCAT	430
*UFGT*	CATGTGGCCGTCCTRGCCTTYCC	GAGGAGCCCATTCCACCACA	950

The full lengths or longer fragments of these genes were obtained after 3′ and 5′-RACE. And then the sequences obtained were compared with known sequences from other species using NCBI Blast server (Table 3). Genbank accession codes of the six isolated genes were listed in Table 3. The coding region of *LcCHS* was **1279 **bp long, encoding a deduced 393-amino acid sequence. *LcCHS* was 81% homologous with the *CHSs* from *Dictamnus albus*. The coding sequence of *LcF3H* (**1196** bp) which was deduced to encode a **364**-amino acid sequence, showed 97% identity with that of *Dimocarpus longan*. The fragments for *LcCHI* (912 bp), *LcDFR* (1017 bp), *LcANS* (1074 bp) and *LcUFGT* (1560 bp) of ‘Nuomici’ showed 86%, 79%, 95% and 67% identities to those of other plants excluding *Arabidopsis*, respectively. In the case of UFGT, the similarity was the lowest, which was in agreement with previous reports in other plants [15], [21]. The main anthocyanin identified in litchi pericarp is cyanidin-3-rutinoside, while the cyanidin-3-glucoside level is very low and no cyanidin-3-galactoside can be detected (Fig. 3). This suggests that the key enzyme catalyzing the conversion of anthocyanidin to anthocyanin in litchi may be neither UDP glucose:flavonoid 3-*O*-glucosyltransferase (UFGluT) nor UDP galactose:flavonoid 3-*O*-galactosyltransferase (UFGalT). Further characterization of substrate and sugar specificity of litchi UFGT will be necessary to investigate.

**Table 3:** Homologies based on nucleotide sequences for anthocyanin biosynthetic genes isolated from litchi cv. Nuomici.

Gene	GenBank number	Top *Arabidopsis* BLAST match	Top BLAST match excluding *Arabidopsis*	Homology (%)
*LcCHS*	GU288820.1	AT5G13930 ATCHS TT4	AJ850132.1 *CHS*1 *Dictamnus albus*	76^a^, 81^b^
*LcCHI*	HQ402910	AT3G55120 ATCHI TT5	FJ887897.1 *CHI Citrus unshiu*	70^a^, 86^b^
*LcF3H*	HQ402911	AT3G51240 ATF3H TT6	EF468104.1 *F3H Dimocarpus longan*	77^a^, 97^b^
*LcDFR*	HQ402912	AT5G42800 ATDFR TT3	AY519363.1 *DFR Citrus sinensis*	73^a^, 79^b^
*LcANS*	HQ402913	AT4G22880 ATANS TT18	FJ479616.1 *ANS Dimocarpus longan*	73^a^, 95^b^
*LcUFGT*	HQ402914	AT5G17050 ATUFGT	FJ169463.1 *UFGT Vitis amurensis*	64^a^, 67^b^

a % Similarity to *Arabidopsis.*

b % Similarity to other plant sequence.

### Expression of six anthocyanin biosynthetic genes in different fruit color phenotype litchis

**Figure 4:**
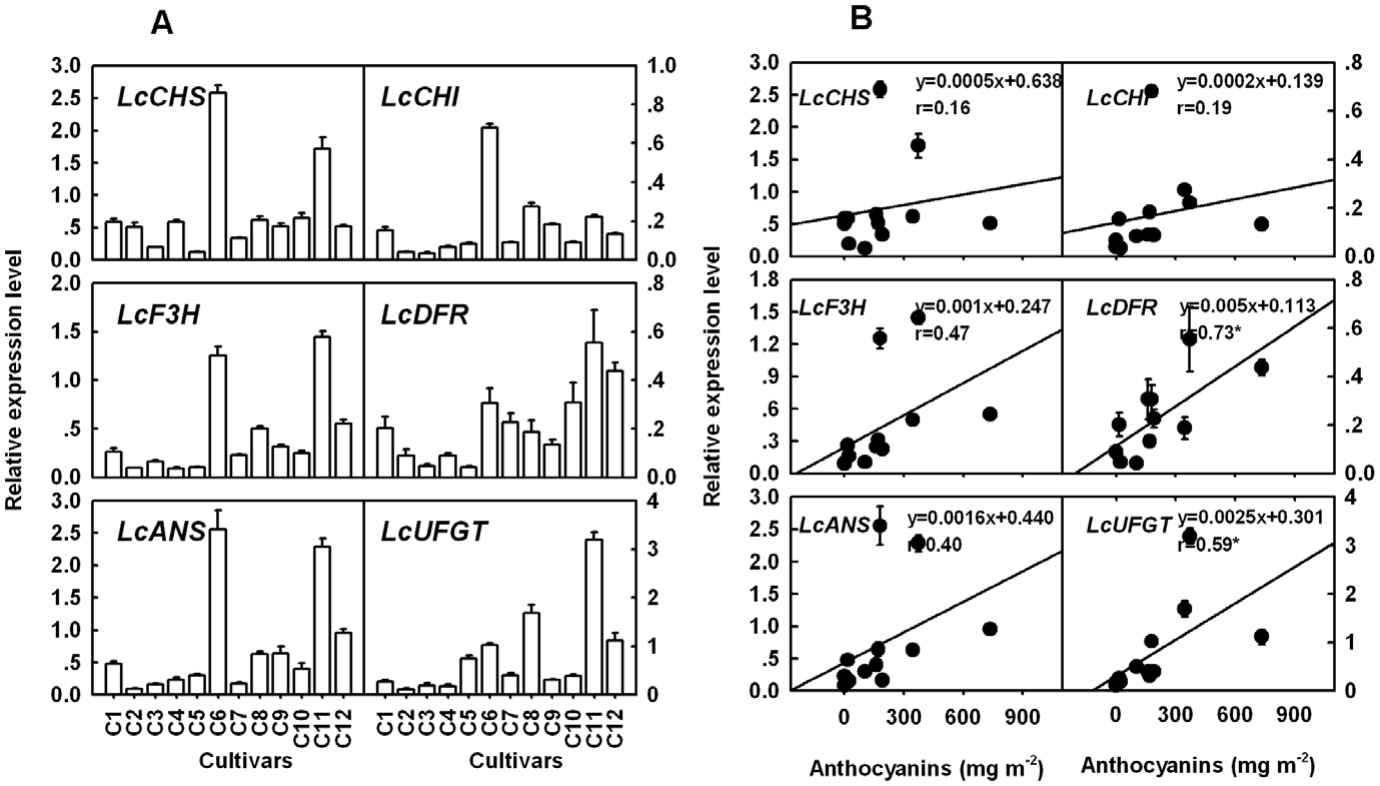
Expression analysis of anthocyanin biosynthetic genes (A) and their correlationship with anthocyanin concentration (B) in the pericarp of twelve litchi cultivars. *Lcactin* gene was used to normalize expression of the genes under identical conditions. The vertical bars represent standard error of three replicates. C1 to C12 are different cultivars explained in Figure 1. Relative coefficient r with ‘*’ indicated significantly correlated at the level of P<0.05. Results of ANOVA test are presented in Table S2.

To elucidate the molecular mechanisms for red coloration in the pericarp of litchi, the transcripts of anthocyanin structural genes were examined in non-red, unevenly red and evenly red cultivars of litchi at maturity. Primers for real-time PCR analysis and product size were shown in Table 4. Basically, in non-red varieties, ie. ‘Kuixingqingpitian’, Xingqiumili’, ‘Yamulong’ and ‘Yongxing No. 2′, the expression of six structural genes, especially the late structural genes from F3H to UFGT was much lower than in the red cultivars (Fig. 4 A). The expression patterns of the early genes, ie. *LcCHS, LcCHI*, *LcF3H*, *LcDFR* and *LcANS*, displayed striking difference between two unevenly red cultivars, ‘Feizixiao’ and ‘Sanyuehong’. The former showed much lower expression levels than the later, though they contained comparable anthocyanins. However, they had comparable *LcUFGT* expression level.

**Table 4:** Primers for real-time PCR analysis.

Gene	Forward primer (5′ to 3′)	Reverse primer (5′ to 3′)	Product (bp)
*LcCHS*	GACATTGTGGTGGTGGAGGT	TATTTAGCGAGACGGAGGAC	242
*LcCHI*	CGGAGTTTACTTGGAGGATGT	CAGTGACCTTCTCAGAGTATTG	185
*LcF3H*	GGTGGATAGATGTGACAAAGGAGT	GGTTGTGGGCATTTTGGATAGTA	169
*LcDFR*	ATAAAGCCAACTATCAATGGGAT	AGCCCATATCACTCCAGCAAGT	160
*LcANs*	AGGAAGTTGGTGGTCTGGAAG	CCGTTGCTGAGGATTTCAATGGTG	274
*LcUFGT*	GCCACCAGCGGTTCCTAATA	ATGCCTCTGCTACTGCTACAATCT	134
*Lcactin*	GTGGTTCTACTATGTTCCCTG	CTCGTCGTACTCATCCTTTG	191

Detail information about cloning of *LcCHS*, *LcCHI*, *LcF3H*, *LcDFR*, *LcANS*, *LcUFGT* and *Lcactin* is presented in Table S5 and Table S6.

To clarify the relationship between the expression of anthocyanin biosynthetic genes and anthocyanin accumulation, their correlations were calculated among the 12 tested cultivars (Figure 4 B). The expression levels of these genes especially the late structural genes from *LcF3H* to *LcUFGT* and anthocyanin concentration in the pericarp displayed positive correlations. Significant relations were observed between the expression of *LcDFR* (r = 0.73) and *LcUFGT* (r = 0.59) and anthocyanin concentration.

### Effects of ABA and CPPU on coloration and anthocyanin biosynthetic gene expression

**Figure 5:**
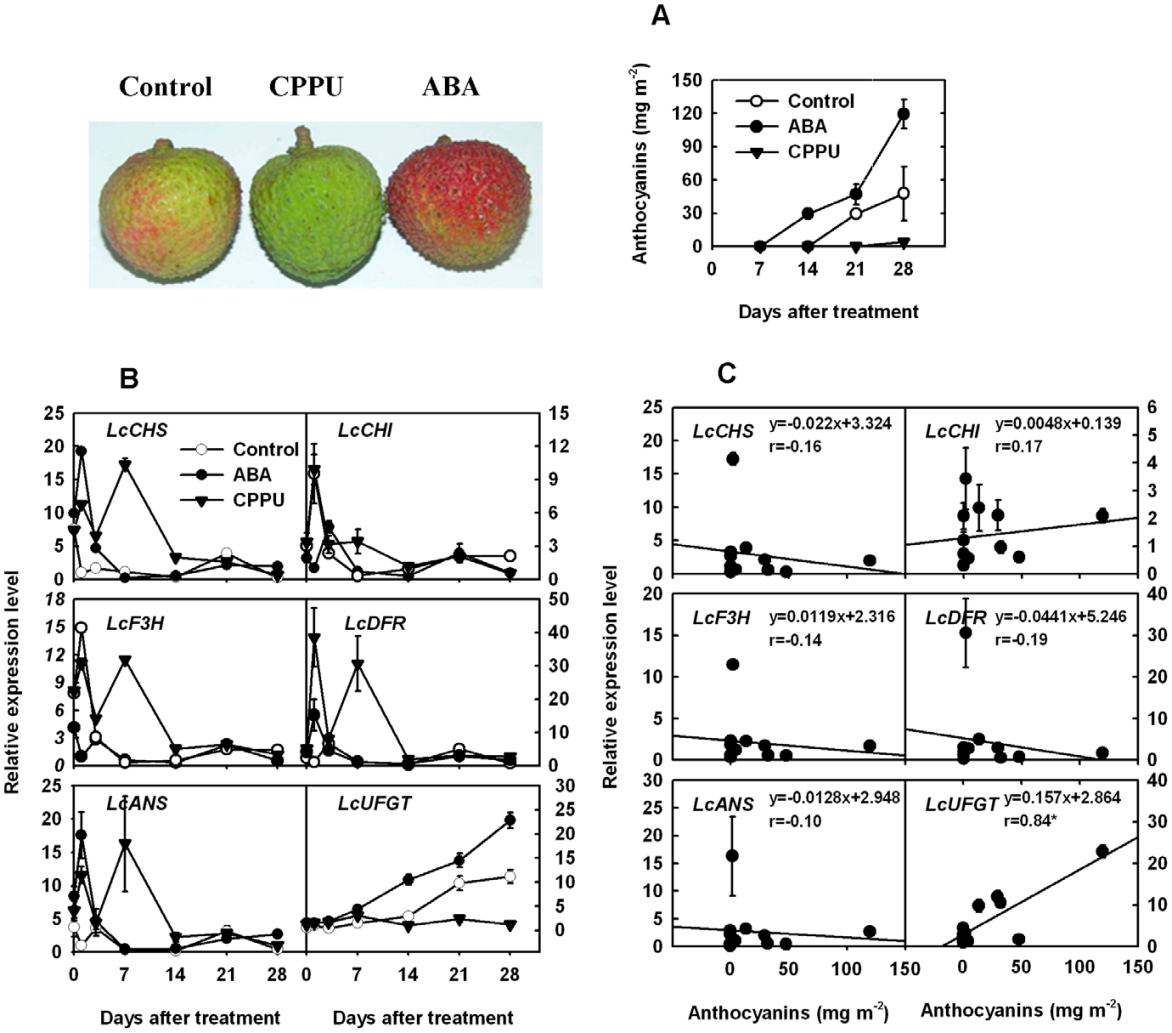
Effects of ABA and CPPU on pigmentation and expression of anthocyanin biosynthetic genes. A) difference in fruit color and anthocyanin concentration in pericarp of ‘Feizixiao’ treated with ABA and CPPU; B) effects of ABA and CPPU on the expression of anthocyanin biosynthetic genes in the pericarp cv. Feizixiao; C) correlations between anthocyanin concentration and expression of anthocyanin biosynthetic genes in pericarp of ‘Feizixiao’. *Lcactin* gene was used to normalize expression of the genes under identical conditions. The vertical bars represent standard error of three replicates. Relative coefficient r with ‘*’ indicated significantly correlated at the level of P<0.05. Results of ANOVA test are presented in Table S3.

Fruit color, concentrations of anthocyanins and the expression of anthocyanin biosynthetic genes in response to ABA and CPPU treatments were showed in Figure 5 A–C. ABA improved while CPPU delayed coloration of litchi cv. Feizixiao (Fig. 5 A), suggesting the biosynthesis of anthocyanins in the pericarp of ‘Feizixiao’ was accelerated by ABA while retarded by CPPU. In the pericarp of the control fruit, no anthcocyanin accumulation occurred before 14 days after treatment (DAT), but it was notably induced at 21 DAT, resulting in a 3.6-fold increase from 21 to 28 DAT (13.4 to 47.7 mg m^−2^). In the pericarp of ABA treatment, no significant accumulation of anthocyanins was detectable at 7 DAT; thereafter, a rapid accumulation from 7 to 28 DAT occurred, resulting in a 2.5-fold higher level of anthocyanins (119.5 mg m^−2^) than the control at harvest. In the pericarp of CPPU treatment, however, no notable anthocyanin accumulation occurred until 28 DAT, resulting a concentration which was less than one tenth of the control.

The expression patterns of the six tested genes were similar in the pericarp of ‘Feizixiao’ from 0 to 28 DAT with the exception of *LcUFGT* (Fig. 5C). The expression of *LcCHS, LcCHI, LcF3H, LcDFR* and *LcANS* was low in the pericarp of the control throughout experimental period. The expression of all these five genes was up-regulated during 0 to 14 DAT in CPPU treatment and 0 to 3 DAT in ABA treatment. The expression pattern of *LcUFGT* was found paralleling with anthocyanin accumulation among treatments. Expression of *LcUFGT* was detected in all of the red pericarps, but not in any of the non-red pericarps. Its expression was not detectable before 14 DAT, after which there was a notable expression in the control. In the pericarp with CPPU treatment, the expression of *LcUFGT* was hardly detectable during the whole experiment period, while a steady increase of *LcUFGT* expression was observed in ABA treatment.

We correlated the expression of six anthocyanin biosynthetic genes to anthocyanin concentrations in different pericarp parts with different color in ‘Feizixiao’. Regression curves and correlation coefficients are shown in Fig. 5 C. Only the expression of *LcUFGT* was found significantly correlated with anthocyanin concentration (r = 0.84).

### Effects of bagging and debagging on anthocyanin accumulation and anthocyanin biosynthetic gene expression

**Figure 6:**
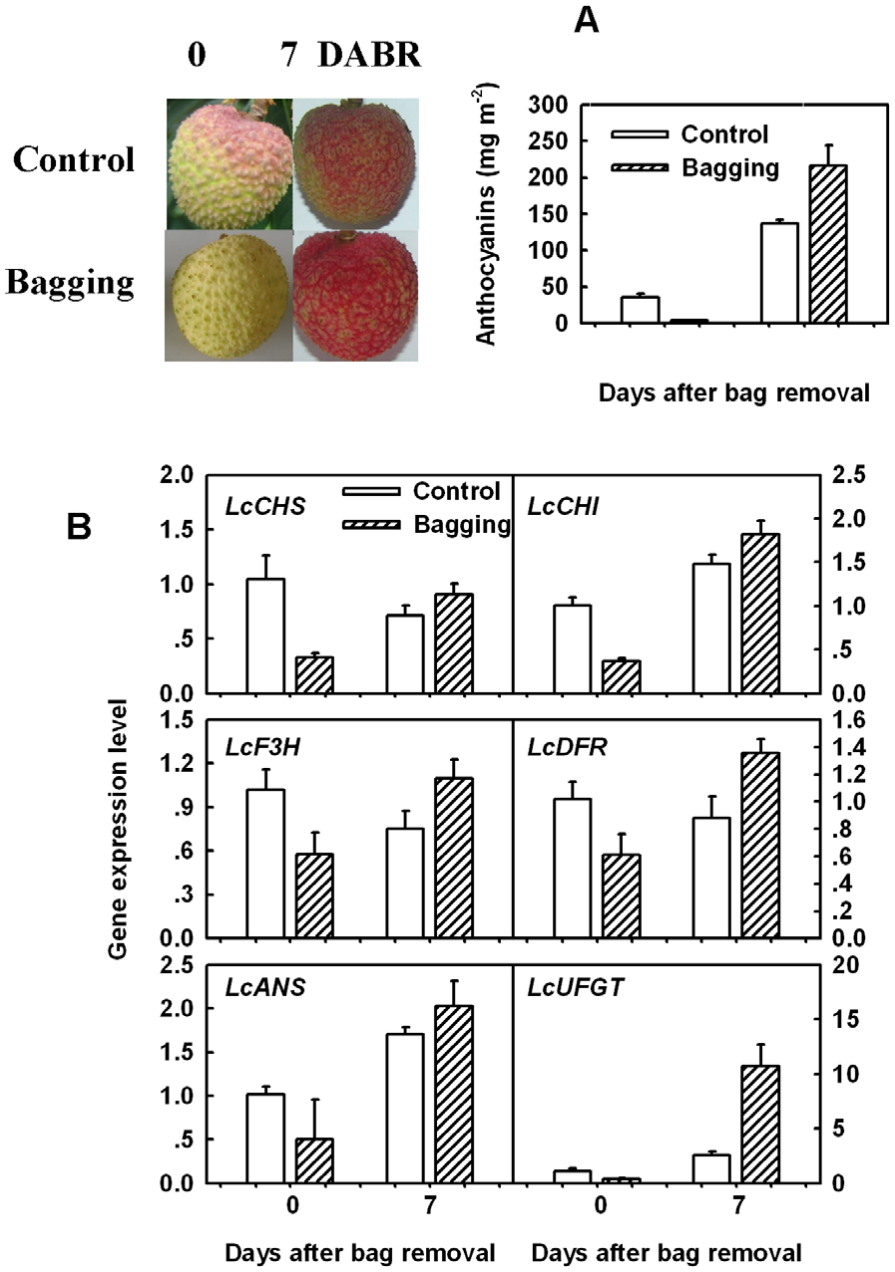
Effects of bagging and bag removal on pigmentation and expression of anthocyanin biosynthetic genes. A) difference in fruit color and anthocyanin concentration in fruit of ‘Feizixiao’ after bagging and bag removal; B) expression analysis of anthocyanin biosynthetic genes in the pericarp of ‘Feizixiao’ after bagging and bag removal. The vertical bars represent standard error of three replicates.

Bagging and bag removal were employed to study the effects of illumination on anthocyanin accumulation and the expression of anthocyanin biosynthetic genes (Fig. 6). Both color development and anthocyanin accumulation were greatly inhibited by bagged in the pericarp of ‘Feizixiao’, with an anthocyanin concentration being less than 10% of that of non-bagged fruit (Fig 6A). But significant anthocyanin accumulation occurred after bag removal. The concentration of anthocyanins increased by 70 times in bagged fruit at 7 days after bag removal, which was about 50% higher than that in the control. This result is consistent with previous studies on apple, pear, and peach, which indicated that sunlight exposure enhanced anthocyanin accumulation [22], [23], [24].

The expression of all anthocyanin biosynthetic genes was possibly inhibited by the bagging treatment and stimulated by bag removal, indicating that sufficient light was essential for expression of the anthocyanin biosynthetic genes. In a study of ‘Cripps’ Red' apples, exposure of bagged fruit to sunlight induced anthocyanin synthesis and the synthesis of anthocyanins correlated with an increase in transcript levels of flavonoid pathway genes [22]. In the present study, the six genes tested were all up-regulated after exposure to sunlight (Fig. 6 B). Among them, *LcUFGT* was most concurrent with anthocyanin accumulation, where low expression level was found particularly in bagged fruit at color break stage and a sharp increase after debagging.

## Discussion

In this study, we demonstrated that a wide range of variability among litchi cultivars in their concentrations of anthocyanins and chlorophylls and chromatic parameters at fruit maturity (Table 1, Fig. 2). Anthocyanins imparted litchi fruit red hues, while green fruit owed their color to chlorophylls. Hue angle correlated negatively with the total anthocyanin concentration (r = −0.78), but positively with chlorophyll concentration (r = 0.86) in the pericarp of litchis (Fig. 2). Generally, the same anthocyanins were present in the red cultivars with similar relative levels (Fig. 3A). The dominant anthoycanin in litchi pericarp was putatively identified as cyanidin-3-rutinoside (>93%), as previously reported by Zhang et al.in cv. Huaizhi [6].

Six genes encoding the anthocyanin biosynthesis enzymes namely *LcCHS, LcCHI, LcF3H, LcDFR, LcANS* and *LcUFGT* were isolated from the pericarp of ‘Nuomici’. These genes were highly homologous, based on BLAST matches, to those from citrus, grape and longan (Table 3). Anthocyanin accumulation was positively correlated with the expression of four anthocyanin biosynthetic genes (*LcF3H, LcDFR, LcANS* and *LcUFGT*) in pericarp of litchi (Fig. 4). The expression of these genes in non-red cultivars, ‘Kuixingqingpitian’, ‘Xingqiumili’, ‘Yamulong’ and ‘Yongxing No. 2′, was weak, whereas in the red one, ‘Feizixiao’, ‘Sanyuehong’, ‘Meiguili’, ‘Baila’, ‘Guiwei’, ‘Nuomici’ and ‘Guinuo’, it was notable. This result suggests that late anthocyanin biosynthetic pathway genes were coordinately expressed in red colored pericarp of litchi, which indicates that alterations of regulating genes may have occurred in these cultivars resulting in decreased synthesis of certain enzymes of the pathway, preventing the accumulation of anthocyanins.

In the present study, we found that the expression of the late genes in anthocyanin synthesis pathway, *LcDFR* and *LcUFGT* in particular, were consistent with the degree of anthocyanin concentration in different color genotypes of litchi. Similar results were also reported in apples [10], [12], [13] and Chinese bayberry [15], indicating that the multiple late genes determined the differential anthocyanin accumulation among different genetypes. The results differed from those reported in grapes where *UFGT* was found the only gene that made the difference in coloration between white type and its red sport [8], [9]. Hence, the different results might be related to the difference in genetic background of the materials studied.

Some enzymes involved in the anthocyanin biosynthetic pathway were studied during development or exogenous stimulus. Lister et al. reported that the activities of CHI and UGFalT in ‘Splendour’ apple were correlated with anthocyanin accumulation during fruit ripening [25]. In ‘Delicious’ and ‘Ralls’ apples exposed to light, CHS activity was not positively correlated with anthocyanin accumulation, whereas UFGalT was positively correlated with anthocyanin accumulation [26]. Moreover, they found that the rapid accumulation of anthocyanins was correlated with an increase in DFR activity in ‘Delicious’ apple [27]. These physiological studies show modification of anthocyanin accumulation by factors beyond the genetic background. In the present study, we investigated developmental changes in the expression of anthocyanin pathway genes and examined their response to growth regulators and illumination conditions (Fig 5, 6). Expression of *LcUFGT* was not detected in any of the green pericarp either before color break or after CPPU application. Hence it appears to be independent of the expression of the other flavonoid synthetic genes in the pericarp of red litchi cv. Feizixiao.

The encoded enzyme UFGT catalyzes the glycosylation of the unstable anthocyanidin aglycones into stable anthocyanins. Only the expression of UFGT was significantly positively correlated with anthocyanin concentration in the pericarp of ‘Feizixiao’ (Fig. 5 C). Our previous studies on the activities of enzymes in anthocyanin biosynthesis including PAL, CHI, DFR and UFGT in the pericarp of ‘Feizixiao’ during fruit development and in response to bagging and growth regulator dipping treatments revealed that only the activity of UFGT was in parallel with the changes in anthocyanin concentration [28]. In the present study, ABA treatment at about one month before commercial harvest enhanced, whereas CPPU treatment at the same date inhibited the expression of *LcUFGT* as well as anthocyanin synthesis (Fig. 5). Accumulation of anthocyanin was also induced and the structural genes in flavonoid pathways were up-regulated in berry skin of the Cabernet Sauvignon grape by ABA application [29]. Cluster bagging inhibited, while bag removal increased both the expression of UFGT and anthocyanin accumulation (Fig. 6). All these results suggest that UFGT was the limiting factor to anthocyanin biosynthesis in the pericarp of ‘Feizixiao’.

The predominant role of UFGT in the coloration of a given red litchi cultivar suggest that *LcUFGT* expression was under a different regulatory regime from the other flavonoid synthetic genes. UFGT could be expressed either synchronously with or independent from other flavonoid synthetic genes.

## Materials and Methods

### Plant material and treatments

Fruit samples of selected twelve litchi cultivars based on their fruit color phenotypes, including four non-red skin cultivars ‘Kuixingqingpitian’, ‘Xinqiumili’, ‘Yamulong’, and ‘Yongxing No. 2′, two unevenly red cultivars ‘Feizixiao’ and ‘Sanyuehong’ and six evenly red cultivars ‘Meiguili’, ‘Baila’,‘Baitangying’, ‘Guiwei’, ‘Nuomici’ and ‘Guinuo’, were taken from Haikou, Hainan province, China and experimental orchard of South China Agricultural University in Guangzhou, Guangdong, China (as shown in Fig. 1). Thirty exposed fruit for each cultivar were picked randomly at commercial maturity. The sampling date, average fruit weight, aril total soluble solid and titratable acid of twelve litchi cultivars are listed in Table S4. After color parameter measurements, pericarp discs were sampled between 10∶00 to 11∶00 am, frozen in liquid N_2_, and stored at −80°C for RNA extraction and other analyses.

The growth regulator applications were carried out 4 weeks before harvest. Triplicate lots from 3 trees of cv. Feizixiao grown in the experimental orchard of South China Agricultural University were sprayed with abscisic acid (ABA, 25 mg l^−1^), forchlorofenron (CPPU, 4 mg l^−1^) and tap water (Control), respectively. After color measurement, pericarp discs were sampled on the day of growth regulator spraying (day 0) and 1, 3, 7, 14, 21 and 28 days after treatments.

In the bagging experiments, three trees of cv. Feizixiao were allotted. Ten clusters existing in different parts of the canopy of each tree were bagged with double-layer kraft paper bags at one month after full bloom. Bags were removed at color break. Clusters in similar development stage grown near the treated ones were served as the control. After color measurements, pericarp discs were sampled on the day of bag removal and on the 7th day after bag removal. All samples were frozen in liquid nitrogen, and stored at −80°C until use.

### Color analyses

The pericarp color variables were measured on 15 fruit samples immediately after picking. L*, a*, and b* values was measured randomly with a Konica Minolta CR-10 Chroma Meter (Minolta, Japan) on the site opposite to the fruit suture. The lightness coefficient ‘L*’, represents brightness and darkness, ‘a*’ value represents greenish and redness as the value increases from negative to positive, and ‘b*’ represents bluish and yellowish. Hue angle (h*) were calculated according to the following equations [30], [31]:

h* = ATAN(b/a)/6.2823*360 when a*≥0 and b*≥0 and h* = ATAN(b/a)/6.2823*360+180 when a*<0 and b*>0.

### Determination of pigments

Total anthocyanins were determined according to the method developed by Fuleki and Francis [32] which involves the measurement of the absorbance at 520 nm on samples diluted with pH 1.0 and 4.5 buffers. Four peel discs (2 cm^2^) were extracted with methanol/water/HCl (3 ml, 85∶12∶3, v/v) for four hours at room temperature at dark. Peel chlorophylls and caroteniods were measured according to Arnon [33].

### HPLC analysis of anthocyanins

Anthocyanins were extracted as above mentioned in anthocyanin determination using a solvent containing methanol : water : HCl (85 ∶ 12 ∶ 3, v/v). The supernatants were filtered through a 0.45 µm Millipore™ filter before used. Anthocyanins in the samples were analyzed using a HP1200-DAD system (Agilent Technologies, Waldbronn, Germany). Detection was performed at 510 nm. A NUCLEODUR^®^ C18 column (250 mm×4.6×mm) (Pretech Instruments, Sollentuna, Sweden) was used for separation at 35°C and eluted using a mobile phase consisting of solvent A (1.6% formic acid in methanol) and solvent B (1.6% formic acid in water) at a flow rate of 1.0 ml min^−1^. The elution program was followed the procedure described by Wu and Prior [34] with some modifications. Solvent A was 15% initially and increased linearly in steps to 20% at 5 min, 28% at 10 min, 40% at 28 min to 40 min.

### RNA extraction and cDNA synthesis

Total RNA was extracted from pericarp tissues using the RNA_OUT_ kit (Tiandz, Beijing). DNase I (TaKaRa, Japan) was added to remove genomic DNA [35] and RNase-free columns (Tiandz, Beijing) were used for purifying total RNA. The concentration of total RNA was measured by absorbance at 260 nm using BioPhotometer Plus (Eppendorf, Germany), and the integrity and quality of the RNA was checked using agarose gel electrophoresis and A_260/280_ ratio. Subsequently, first-strand cDNA was synthesized from total RNA (2 µg) using oligo(dT) primers following the manufacturer's instructions of PrimeScript™ RT-PCR Kit (TaKaRa, Japan).

### Cloning of anthocyanin biosynthetic genes

Degenerate primers were designed based on the highly conserved peptide regions of CHS, CHI, F3H, DFR, ANS and UFGT (Table 2). The cDNAs encoding these proteins were amplified by PCR using these degenerate primers. cDNAs synthesized from mature pericarp of cv. Nuomici were used as PCR templates. Amplified PCR products of appropriate length were cloned into T/A cloning vector pMD^®^ 20-T (TaKaRa, Japan) and then transformed into *E.coli* DH5α Max Efficiency chemically competent cells (TaKaRa, Japan). Plasmid DNA isolated from positive *E.coli* cells was digested with *Eco*R I, and the inserted DNA was sent to Beijing Genomics Institute for sequencing.

Rapid amplification of cDNA ends (RACE) was performed to obtain the 3′and 5′ ends of these six genes in anthocyanin biosynthetic pathway from mature pericarp cv. Nuomici using 3′ -Full RACE Core Set Ver.2.0 and 5′ RACE Kit (TaKaRa, Japan). Full-length or partial-length cDNA sequences encoding CHS, CHI, F3H, DFR, ANS and UFGT enzymes are available in the GenBank nucleotide database.

### Sequence analysis

Analysis of CHS, CHI, F3H, DFR, ANS, and UFGT sequences and comparing them with known sequences was carried out using NCBI Blast server [36]. Multiple sequence alignment was performed using ClustalX 1.83 (http://www.ebi.ac.uk) [37].

### Quantitative real-time PCR analysis

Isolation of total RNA from the pericarp of litchis and synthesis first strand cDNA were performed as described above. The transcript levels of *LcCHS*, *LcCHI*, *LcF3H*, *LcDFR*, *LcANS*, and *LcUFGT* were analysed using quantitative real-time PCR (RT-qPCR) with THUNDERBIRD qPCR Mix (TOYOBO, Japan) and ABI 7500 Real-Time PCR Systems (Applied Biosystems, USA), according to the manufacturers' instructions. Each reaction (final volume, 20 µl) contained 10 µl 2×SYBR® qPCR Mix (TOYOBO), 0.04 µl 50×ROX reference dye, 1 µl of each the forward and reverse primers (0.25 µM), 2 µl of the cDNA template (corresponding to 50 ng of total RNA), and 7 µl of RNase-free water. The reaction mixtures were heated to 95°C for 30 s, followed by 35 cycles at 95°C for 10 s, 55°C for 15 s, and 72°C for 30 s. A melting curve was generated for each sample at the end of each run to ensure the purity of the amplified products.

All gene-specific primers from the identified genes for real-time PCR were designed using a Primer 5.0 program (PREMIER Biosoft International, Canada) (Table 4). Each assay using the gene-specific primers amplified a single product of correct size with high PCR efficiency (90%–110%) [38]. Among seven frequently used candidate reference genes, actin gene (GenBank accession number:HQ615689) was stably expressed in varieties and fruit developmental stage according to a study on selection of reliable reference genes for expression study by qRT-PCR in litchi [39]. Actin gene also exhibited expression stability in ABA and CPPU treatments (See Table S7). All qRT-PCR reactions were normalized using Ct value corresponding to the actin gene. The relative expression levels of target genes were calculated with formula 2^−▵▵CT^ [40]. Values reported represent the average of three biological replicate.

### Statistical analysis

Statistical analyses were performed using the statistical package DPS v3.0 (Hangzhou, China). Duncan multiple range test was used to determine significance of color parameter differences at the 5% level. Pearson correlation coefficients were calculated and a two-tailed test was used to determine significance at the 5% level.

## Supporting Information

Figure S1 HPLC elution profile of anthocyanins from pericarp of full red litchi cultivars.
(DOC)

Table S1 Results of ANOVA test for L*, a*, b*and h* among twelve cultivars.
(XLS)

Table S2 Results of ANOVA test on relative coefficients between anthocyanin concentration and gene expression level in the pericarp of twelve cultivars.
(DOC)

Table S3 Results of ANOVA test on relative coefficients between anthocyanin concentration and gene expression level in the pericarp of different pigmentation pericarp of ‘Feizixiao’.
(DOC)

Table S4 Sampling date, fruit weight, total soluble solid and titratable acid of litchis at maturity.
(DOC)

Table S5 Cloning of *LcCHS*, *LcCHI*, *LcF3H*, *LcDFR*, *LcANS* and *LcUFGT*.
(DOC)

Table S6 Cloning and identification of *LcActin.*
(DOC)

Table S7 Evaluating the expression stability of reference genes.
(DOC)
