# Supplementary material for: Differential Expression of Anthocyanin Biosynthetic Genes in Relation to Anthocyanin Accumulation in the Pericarp of Litchi Chinensis Sonn
Source: PLoS One. 2011 Apr 29;6(4):e19455. doi: 10.1371/journal.pone.0019455 (PMC3084873; doi:10.1371/journal.pone.0019455)
Supplement: Figure S1 — HPLC elution profile of anthocyanins from pericarp of full red litchi cultivars. (DOC) [file pone.0019455.s001.doc]

Figure S1 HPLC elution profile of anthocyanins from pericarp of full red litchi cultivars.

| ‘Meiguili’ | ‘Baila’ |
| --- | --- |
| 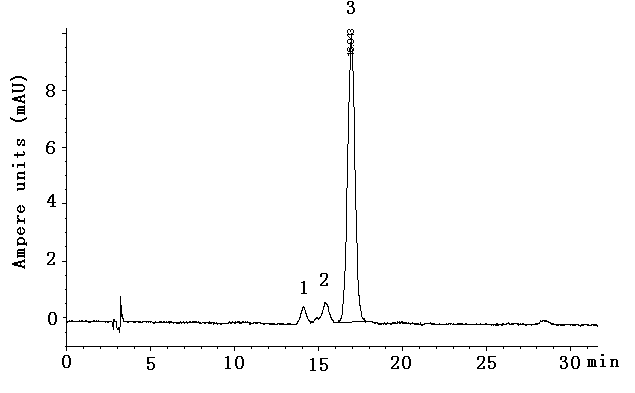 | 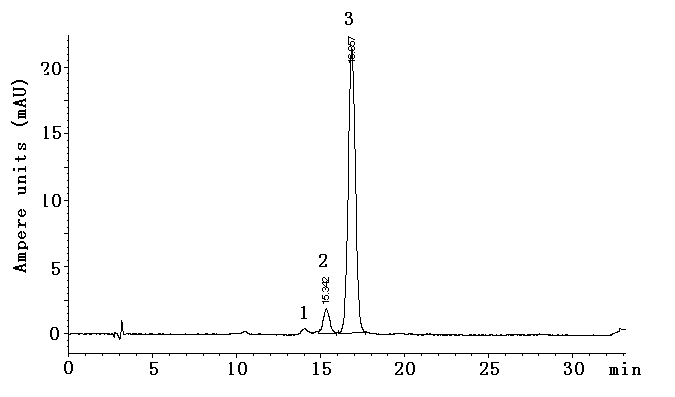 |
| ‘Baitangying’ | ‘Guiwei’ |
| 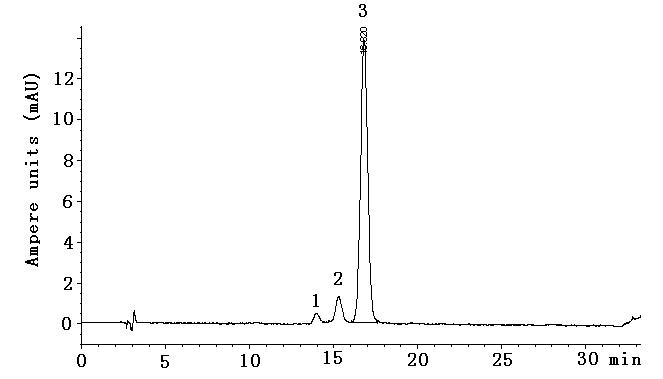 | 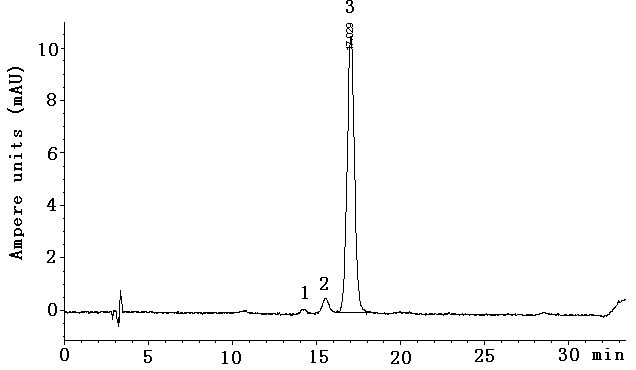 |
| ‘Nuomici’ | ‘Guinuo’ |
| 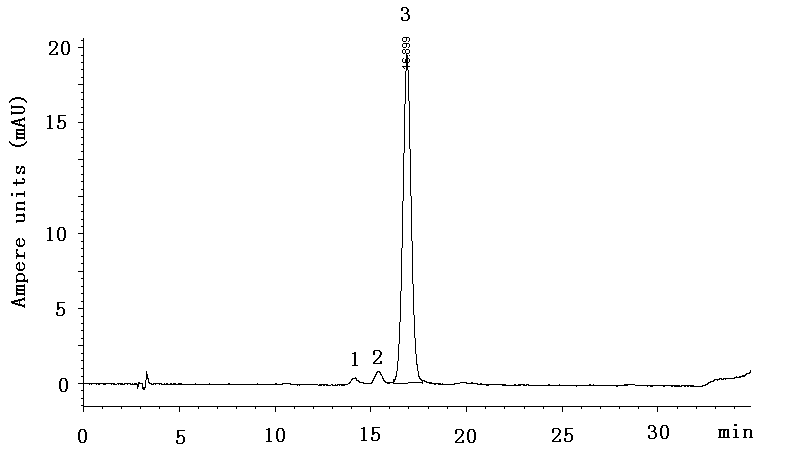 | 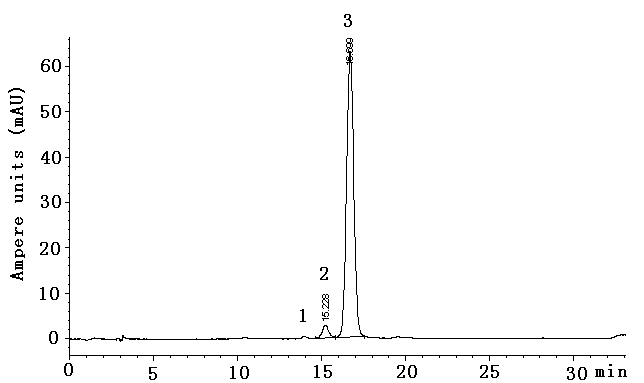 |

All full red cultivars examined contained the same three peaks. Peak 1 to 3 were cyanidin (Peak 1), cyanidin-3-glucoside (Peak 2), cyanidin-3-rutinoside (Peak 3) respectively, which were putatively identiﬁed through the comparison of retention time and spectrum characters with the published data (Lee and Wicker,1991;Rivera-López et al,1999; Zhang et al., 2004).
