# Supplementary material for: Differential Expression of Anthocyanin Biosynthetic Genes in Relation to Anthocyanin Accumulation in the Pericarp of Litchi Chinensis Sonn
Source: PLoS One. 2011 Apr 29;6(4):e19455. doi: 10.1371/journal.pone.0019455 (PMC3084873; doi:10.1371/journal.pone.0019455)
Supplement: Table S2 — Results of ANOVA test on relative coefficients between anthocyanin concentration and gene expression level in the pericarp of twelve cultivars. (DOC) [file pone.0019455.s003.doc]

**Table S2 Results of ANOVA test on relative coefficients between anthocyanin concentration and gene expression level in the pericarp of twelve cultivars**

| Cultivars | anthocyanins | chs |
| --- | --- | --- |
| Kuixingqingpitian | 14.78 | 0.59 |
| Xingqiumili | 0.00 | 0.51 |
| Yamulong | 21.63 | 0.19 |
| Yongxing No.2 | 0.00 | 0.58 |
| Feizixiao | 103.70 | 0.12 |
| Sanyuehong | 178.65 | 2.58 |
| Meiguili | 190.00 | 0.33 |
| Baila | 344.53 | 0.61 |
| Baitangying | 169.43 | 0.51 |
| Guiwei | 159.76 | 0.64 |
| Nuomici | 371.55 | 1.71 |
| Guinuo | 733.50 | 0.51 |


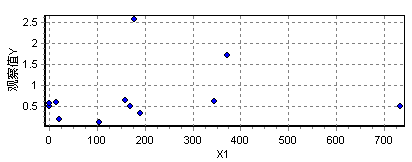


| Results |  | | | | | | |  |
| --- | --- | --- | --- | --- | --- | --- | --- | --- |
| Variables | Means | Standard deviations | Variance Inflation Factors |  |  |  |  | |
| x1 | 190.6267 | 211.5723 | 1.0000 |  |  |  |  | |
| y | 0.7414 | 0.7017 |  |  |  |  |  | |
|  |  |  |  |  |  |  |  | |
| correlation coefficient |  |  |  |  |  |  |  | |
|  | x1 | y |  |  |  |  |  | |
| x1 | 1.0000 | 0.6138 |  |  |  |  |  | |
| y | 0.1625 | 1.0000 |  |  |  |  |  | |
|  |  |  |  |  |  |  |  | |
|  | Variance analysis |  |  |  |  |  |  | |
| Source of variance | Sum of squares | df | Mean squares | F-value | p-value |  |  | |
| Regression | 0.1430 | 1 | 0.1430 | 0.2712 | 0.6138 |  |  | |
| Residual | 5.2737 | 10 | 0.5274 |  |  |  |  | |
| total | 5.4167 | 11 | 0.4924 |  |  |  |  | |
|  |  |  |  |  |  |  |  | |

| Cultivars | anthocyanins | chi |
| --- | --- | --- |
| Kuixingqingpitian | 14.78 | 0.15 |
| Xingqiumili | 0.00 | 0.04 |
| Yamulong | 21.63 | 0.03 |
| Yongxing No.2 | 0.00 | 0.07 |
| Feizixiao | 103.70 | 0.08 |
| Sanyuehong | 178.65 | 0.68 |
| Meiguili | 190.00 | 0.09 |
| Baila | 344.53 | 0.27 |
| Baitangying | 169.43 | 0.18 |
| Guiwei | 159.76 | 0.09 |
| Nuomici | 371.55 | 0.22 |
| Guinuo | 733.50 | 0.13 |


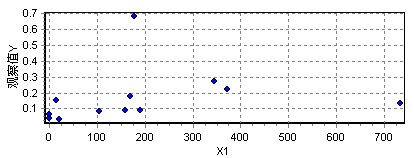


| Results |  | | | | | | |  |
| --- | --- | --- | --- | --- | --- | --- | --- | --- |
| Variables | Means | Standard deviations | Variance Inflation Factors |  |  |  |  | |
| x1 | 190.6267 | 211.5723 | 1.0000 |  |  |  |  | |
| y | 0.1696 | 0.1769 |  |  |  |  |  | |
|  |  |  |  |  |  |  |  | |
| correlation coefficient |  |  |  |  |  |  |  | |
|  | x1 | y |  |  |  |  |  | |
| x1 | 1.0000 | 0.5488 |  |  |  |  |  | |
| y | 0.1926 | 1.0000 |  |  |  |  |  | |
|  |  |  |  |  |  |  |  | |
|  | Variance analysis |  |  |  |  |  |  | |
| Source of variance | Sum of squares | df | Mean squares | F-value | p-value |  |  | |
| Regression | 0.0128 | 1 | 0.0128 | 0.3851 | 0.5488 |  |  | |
| Residual | 0.3313 | 10 | 0.0331 |  |  |  |  | |
| total | 0.3440 | 11 | 0.0313 |  |  |  |  | |

| Cultivars | anthocyanins | | f3h | |  |
| --- | --- | --- | --- | --- | --- |
| Kuixingqingpitian | | 14.78 | | 0.26 | |
| Xingqiumili | | 0.00 | | 0.09 | |
| Yamulong | | 21.63 | | 0.16 | |
| Yongxing No.2 | | 0.00 | | 0.09 | |
| Feizixiao | | 103.70 | | 0.10 | |
| Sanyuehong | | 178.65 | | 1.25 | |
| Meiguili | | 190.00 | | 0.23 | |
| Baila | | 344.53 | | 0.50 | |
| Baitangying | | 169.43 | | 0.31 | |
| Guiwei | | 159.76 | | 0.25 | |
| Nuomici | | 371.55 | | 1.45 | |
| Guinuo | | 733.50 | | 0.55 | |


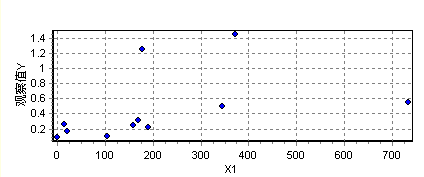


| Results |  | | | | | | |
| --- | --- | --- | --- | --- | --- | --- | --- |
| Variables | Means | Standard deviations | Variance Inflation Factors |  |  |  |  |
| x1 | 190.6267 | 211.5723 | 1.0000 |  |  |  |  |
| y | 0.4367 | 0.4529 |  |  |  |  |  |
|  |  |  |  |  |  |  |  |
| correlation coefficient |  |  |  |  |  |  |  |
|  | x1 | y |  |  |  |  |  |
| x1 | 1.0000 | 0.1274 |  |  |  |  |  |
| y | 0.4654 | 1.0000 |  |  |  |  |  |
|  |  |  |  |  |  |  |  |
|  | Variance analysis |  |  |  |  |  |  |
| Source of variance | Sum of squares | df | Mean squares | F-value | p-value |  |  |
| Regression | 0.4886 | 1 | 0.4886 | 2.7643 | 0.1274 |  |  |
| Residual | 1.7675 | 10 | 0.1767 |  |  |  |  |
| total | 2.2560 | 11 | 0.2051 |  |  |  |  |
|  |  |  |  |  |  |  |  |

| Cultivars | anthocyanins | dfr |
| --- | --- | --- |
| Kuixingqingpitian | 14.78 | 0.20 |
| Xingqiumili | 0.00 | 0.09 |
| Yamulong | 21.63 | 0.05 |
| Yongxing No.2 | 0.00 | 0.09 |
| Feizixiao | 103.70 | 0.04 |
| Sanyuehong | 178.65 | 0.31 |
| Meiguili | 190.00 | 0.23 |
| Baila | 344.53 | 0.19 |
| Baitangying | 169.43 | 0.13 |
| Guiwei | 159.76 | 0.31 |
| Nuomici | 371.55 | 0.55 |
| Guinuo | 733.50 | 0.44 |


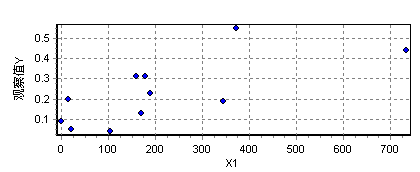


| Results |  | | | | | | |
| --- | --- | --- | --- | --- | --- | --- | --- |
| Variables | Means | Standard deviations | Variance Inflation Factors |  |  |  |  |
| x1 | 190.6275 | 211.5725 | 1.0000 |  |  |  |  |
| y | 0.2192 | 0.1590 |  |  |  |  |  |
|  |  |  |  |  |  |  |  |
| correlation coefficient |  |  |  |  |  |  |  |
|  | x1 | y |  |  |  |  |  |
| x1 | 1.0000 | 0.0071 |  |  |  |  |  |
| y | 0.7297 | 1.0000 |  |  |  |  |  |
|  |  |  |  |  |  |  |  |
|  | Variance analysis |  |  |  |  |  |  |
| Source of variance | Sum of squares | df | Mean squares | F-value | p-value |  |  |
| Regression | 0.1481 | 1 | 0.1481 | 11.3891 | 0.0071 |  |  |
| Residual | 0.1300 | 10 | 0.0130 |  |  |  |  |
| total | 0.2781 | 11 | 0.0253 |  |  |  |  |

| Cultivars | anthocyanins | ans |
| --- | --- | --- |
| Kuixingqingpitian | 14.78 | 0.48 |
| Xingqiumili | 0.00 | 0.09 |
| Yamulong | 21.63 | 0.16 |
| Yongxing No.2 | 0.00 | 0.23 |
| Feizixiao | 103.70 | 0.30 |
| Sanyuehong | 178.65 | 2.55 |
| Meiguili | 190.00 | 0.17 |
| Baila | 344.53 | 0.63 |
| Baitangying | 169.43 | 0.64 |
| Guiwei | 159.76 | 0.40 |
| Nuomici | 371.55 | 2.29 |
| Guinuo | 733.50 | 0.95 |


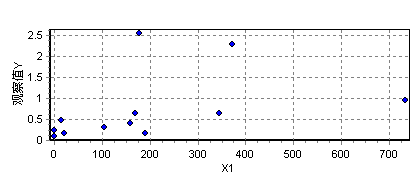


| Results |  | | | | | | |
| --- | --- | --- | --- | --- | --- | --- | --- |
| Variables | Means | Standard deviations | Variance Inflation Factors |  |  |  |  |
| x1 | 190.6275 | 211.5725 | 1.0000 |  |  |  |  |
| y | 0.7408 | 0.8238 |  |  |  |  |  |
|  |  |  |  |  |  |  |  |
| correlation coefficien |  |  |  |  |  |  |  |
|  | x1 | y |  |  |  |  |  |
| x1 | 1.0000 | 0.1944 |  |  |  |  |  |
| y | 0.4026 | 1.0000 |  |  |  |  |  |
|  |  |  |  |  |  |  |  |
|  | Variance analysis |  |  |  |  |  |  |
| Source of variance | Sum of squares | df | Mean squares | F-value | p-value |  |  |
| Regression | 1.2101 | 1 | 1.2101 | 1.9345 | 0.1944 |  |  |
| Residual | 6.2554 | 10 | 0.6255 |  |  |  |  |
| total | 7.4655 | 11 | 0.6787 |  |  |  |  |

| Cultivars | | anthocyanins | | ufgt |
| --- | --- | --- | --- | --- |
| Kuixingqingpitian | 14.78 | | 0.26 | |
| Xingqiumili | 0.00 | | 0.11 | |
| Yamulong | 21.63 | | 0.19 | |
| Yongxing No.2 | 0.00 | | 0.17 | |
| Feizixiao | 103.70 | | 0.50 | |
| Sanyuehong | 178.65 | | 1.02 | |
| Meiguili | 190.00 | | 0.40 | |
| Baila | 344.53 | | 1.69 | |
| Baitangying | 169.43 | | 0.31 | |
| Guiwei | 159.76 | | 0.39 | |
| Nuomici | 371.55 | | 3.19 | |
| Guinuo | 733.50 | | 1.12 | |


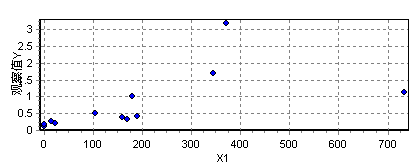


| Variables | Means | Standard deviations | Variance Inflation Factors |  |  |  |  |
| --- | --- | --- | --- | --- | --- | --- | --- |
| x1 | 190.6275 | 211.5725 | 1.0000 |  |  |  |  |
| y | 0.7792 | 0.8967 |  |  |  |  |  |
|  |  |  |  |  |  |  |  |
| correlation coefficient |  |  |  |  |  |  |  |
|  | x1 | y |  |  |  |  |  |
| x1 | 1.0000 | 0.0417 |  |  |  |  |  |
| y | 0.5940 | 1.0000 |  |  |  |  |  |
|  |  |  |  |  |  |  |  |
|  | Variance analysis |  |  |  |  |  |  |
| Source of variance | Sum of squares | df | Mean squares | F-value | p-value |  |  |
| Regression | 3.1202 | 1 | 3.1202 | 5.4507 | 0.0417 |  |  |
| Residual | 5.7245 | 10 | 0.5724 |  |  |  |  |
| total | 8.8447 | 11 | 0.8041 |  |  |  |  |
|  |  |  |  |  |  |  |  |
