# Supplementary material for: Differential Expression of Anthocyanin Biosynthetic Genes in Relation to Anthocyanin Accumulation in the Pericarp of Litchi Chinensis Sonn
Source: PLoS One. 2011 Apr 29;6(4):e19455. doi: 10.1371/journal.pone.0019455 (PMC3084873; doi:10.1371/journal.pone.0019455)
Supplement: Table S3 — Results of ANOVA test on relative coefficients between anthocyanin concentration and gene expression level in the pericarp of different pigmentation pericarp of ‘Feizixiao’. (DOC) [file pone.0019455.s004.doc]

**Table S3 Results of ANOVA test on relative coefficients between anthocyanin concentration and gene expression level in the pericarp of different pigmentation pericarp of ‘Feizixiao’**

| anthocyanins | chs |
| --- | --- |
| 0.00 | 1.13 |
| 0.00 | 0.35 |
| 13.36 | 3.92 |
| 47.74 | 0.26 |
| 0.00 | 0.25 |
| 32.10 | 0.55 |
| 29.44 | 2.12 |
| 119.48 | 1.95 |
| 1.83 | 17.25 |
| 0.00 | 3.29 |
| 0.00 | 2.69 |
| 4.21 | 0.65 |


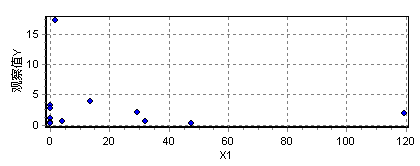


| Variables | Means | Standard deviations | Variance Inflation Factors |  |  |  |  |
| --- | --- | --- | --- | --- | --- | --- | --- |
| x1 | 20.6800 | 35.0575 | 1.0000 |  |  |  |  |
| y | 2.8675 | 4.6988 |  |  |  |  |  |
|  |  |  |  |  |  |  |  |
| correlation coefficient |  |  |  |  |  |  |  |
|  | x1 | y |  |  |  |  |  |
| x1 | 1.0000 | 0.6078 |  |  |  |  |  |
| y | -0.1653 | 1.0000 |  |  |  |  |  |
|  |  |  |  |  |  |  |  |
|  | Variance analysis |  |  |  |  |  |  |
| Source of variance | Sum of squares | df | Mean squares | F-value | p-value |  |  |
| Regression | 6.6331 | 1 | 6.6331 | 0.2808 | 0.6078 |  |  |
| Residual | 236.2367 | 10 | 23.6237 |  |  |  |  |
| total | 242.8698 | 11 | 22.0791 |  |  |  |  |

| anthocyanins | chi |
| --- | --- |
| 0.00 | 0.72 |
| 0.00 | 0.30 |
| 13.36 | 2.37 |
| 47.74 | 0.59 |
| 0.00 | 0.33 |
| 32.10 | 0.96 |
| 29.44 | 2.11 |
| 119.48 | 2.09 |
| 1.83 | 3.43 |
| 0.00 | 1.20 |
| 0.00 | 2.08 |
| 4.21 | 0.56 |


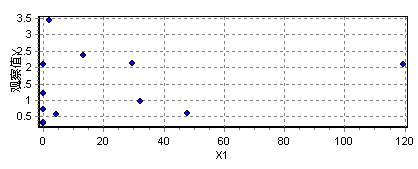


| Variables | Means | Standard deviations | Variance Inflation Factors |  |  |  |  |
| --- | --- | --- | --- | --- | --- | --- | --- |
| x1 | 20.6800 | 35.0575 | 1.0000 |  |  |  |  |
| y | 1.3950 | 0.9962 |  |  |  |  |  |
|  |  |  |  |  |  |  |  |
| correlation coefficient |  |  |  |  |  |  |  |
|  | x1 | y |  |  |  |  |  |
| x1 | 1.0000 | 0.5995 |  |  |  |  |  |
| y | 0.1690 | 1.0000 |  |  |  |  |  |
|  |  |  |  |  |  |  |  |
|  | Variance analysis |  |  |  |  |  |  |
| Source of variance | Sum of squares | df | Mean squares | F-value | p-value |  |  |
| Regression | 0.3118 | 1 | 0.3118 | 0.2940 | 0.5995 |  |  |
| Residual | 10.6049 | 10 | 1.0605 |  |  |  |  |
| total | 10.9167 | 11 | 0.9924 |  |  |  |  |
|  |  |  |  |  |  |  |  |

| anthocyanins | f3h |
| --- | --- |
| 0.00 | 0.61 |
| 0.00 | 0.33 |
| 13.36 | 2.25 |
| 47.74 | 0.54 |
| 0.00 | 0.34 |
| 32.10 | 0.58 |
| 29.44 | 1.74 |
| 119.48 | 1.69 |
| 1.83 | 11.45 |
| 0.00 | 1.81 |
| 0.00 | 2.31 |
| 4.21 | 1.20 |


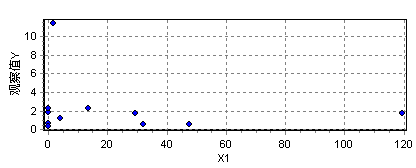


| Variables | Means | Standard deviations | Variance Inflation Factors |  |  |  |  |
| --- | --- | --- | --- | --- | --- | --- | --- |
| x1 | 20.6800 | 35.0575 | 1.0000 |  |  |  |  |
| y | 2.0708 | 3.0431 |  |  |  |  |  |
|  |  |  |  |  |  |  |  |
| correlation coefficient |  |  |  |  |  |  |  |
|  | x1 | y |  |  |  |  |  |
| x1 | 1.0000 | 0.6721 |  |  |  |  |  |
| y | -0.1366 | 1.0000 |  |  |  |  |  |
|  |  |  |  |  |  |  |  |
|  | Variance analysis |  |  |  |  |  |  |
| Source of variance | Sum of squares | df | Mean squares | F-value | p-value |  |  |
| Regression | 1.9004 | 1 | 1.9004 | 0.1901 | 0.6721 |  |  |
| Residual | 99.9649 | 10 | 9.9965 |  |  |  |  |
| total | 101.8653 | 11 | 9.2605 |  |  |  |  |
|  |  |  |  |  |  |  |  |

| anthocyanins | dfr |
| --- | --- |
| 0.00 | 1.23 |
| 0.00 | 0.31 |
| 13.36 | 4.99 |
| 47.74 | 0.75 |
| 0.00 | 1.12 |
| 32.10 | 0.56 |
| 29.44 | 2.95 |
| 119.48 | 1.70 |
| 1.83 | 30.61 |
| 0.00 | 1.94 |
| 0.00 | 3.06 |
| 4.21 | 2.78 |


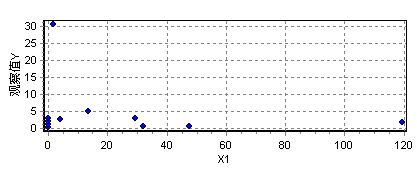


| Variables | Means | Standard deviations | Variance Inflation Factors |  |  |  |  |
| --- | --- | --- | --- | --- | --- | --- | --- |
| x1 | 20.6800 | 35.0575 | 1.0000 |  |  |  |  |
| y | 4.3333 | 8.3814 |  |  |  |  |  |
|  |  |  |  |  |  |  |  |
| correlation coefficient |  |  |  |  |  |  |  |
|  | x1 | y |  |  |  |  |  |
| x1 | 1.0000 | 0.5653 |  |  |  |  |  |
| y | -0.1848 | 1.0000 |  |  |  |  |  |
|  |  |  |  |  |  |  |  |
|  | Variance analysis |  |  |  |  |  |  |
| Source of variance | Sum of squares | df | Mean squares | F-value | p-value |  |  |
| Regression | 26.3831 | 1 | 26.3831 | 0.3535 | 0.5653 |  |  |
| Residual | 746.3433 | 10 | 74.6343 |  |  |  |  |
| total | 772.7265 | 11 | 70.2479 |  |  |  |  |
|  |  |  |  |  |  |  |  |

| anthocyanins | ans |
| --- | --- |
| 0.00 | 0.37 |
| 0.00 | 0.16 |
| 13.36 | 3.15 |
| 47.74 | 0.43 |
| 0.00 | 0.47 |
| 32.10 | 0.55 |
| 29.44 | 2.01 |
| 119.48 | 2.67 |
| 1.83 | 16.30 |
| 0.00 | 2.24 |
| 0.00 | 2.85 |
| 4.21 | 1.01 |


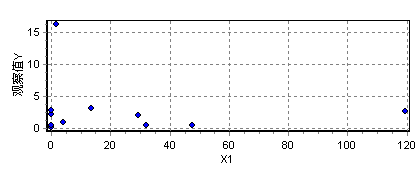


| Variables | Means | Standard deviations | Variance Inflation Factors |  |  |  |  |
| --- | --- | --- | --- | --- | --- | --- | --- |
| x1 | 20.6800 | 35.0575 | 1.0000 |  |  |  |  |
| y | 2.6842 | 4.4247 |  |  |  |  |  |
|  |  |  |  |  |  |  |  |
| correlation coefficient |  |  |  |  |  |  |  |
|  | x1 | y |  |  |  |  |  |
| x1 | 1.0000 | 0.7531 |  |  |  |  |  |
| y | -0.1017 | 1.0000 |  |  |  |  |  |
|  |  |  |  |  |  |  |  |
|  | Variance analysis |  |  |  |  |  |  |
| Source of variance | Sum of squares | df | Mean squares | F-value | p-value |  |  |
| Regression | 2.2284 | 1 | 2.2284 | 0.1046 | 0.7531 |  |  |
| Residual | 213.1271 | 10 | 21.3127 |  |  |  |  |
| total | 215.3555 | 11 | 19.5778 |  |  |  |  |
|  |  |  |  |  |  |  |  |

| anthocyanins | ufgt |
| --- | --- |
| 0.00 | 1.51 |
| 0.00 | 2.90 |
| 13.36 | 9.79 |
| 47.74 | 1.72 |
| 0.00 | 4.44 |
| 32.10 | 10.50 |
| 29.44 | 11.98 |
| 119.48 | 22.82 |
| 1.83 | 3.06 |
| 0.00 | 0.97 |
| 0.00 | 2.38 |
| 4.21 | 1.28 |


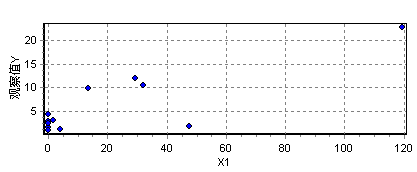


| Variables | Means | Standard deviations | Variance Inflation Factors |  |  |  |  |
| --- | --- | --- | --- | --- | --- | --- | --- |
| x1 | 20.6800 | 35.0575 | 1.0000 |  |  |  |  |
| y | 6.1125 | 6.5573 |  |  |  |  |  |
|  |  |  |  |  |  |  |  |
| correlation coefficient |  |  |  |  |  |  |  |
|  | x1 | y |  |  |  |  |  |
| x1 | 1.0000 | 0.0006 |  |  |  |  |  |
| y | 0.8397 | 1.0000 |  |  |  |  |  |
|  |  |  |  |  |  |  |  |
|  | Variance analysis |  |  |  |  |  |  |
| Source of variance | Sum of squares | df | Mean squares | F-value | p-value |  |  |
| Regression | 333.4763 | 1 | 333.4763 | 23.9037 | 0.0006 |  |  |
| Residual | 139.5081 | 10 | 13.9508 |  |  |  |  |
| total | 472.9844 | 11 | 42.9986 |  |  |  |  |
|  |  |  |  |  |  |  |  |
