# Supplementary material for: Differential Expression of Anthocyanin Biosynthetic Genes in Relation to Anthocyanin Accumulation in the Pericarp of Litchi Chinensis Sonn
Source: PLoS One. 2011 Apr 29;6(4):e19455. doi: 10.1371/journal.pone.0019455 (PMC3084873; doi:10.1371/journal.pone.0019455)
Supplement: Table S4 — Sampling date, fruit weight, total soluble solid and titratable acid of litchis at maturity. (DOC) [file pone.0019455.s005.doc]

**Table S4** Sampling date, fruit weight, total soluble solid and titratable acid of litchis at maturity.

| Cultivars | Sampling date  (Year-Month-Day) | Fruit weight (g) | TSS (^o^Brix) | Titratable acid (%) |
| --- | --- | --- | --- | --- |
| ‘Kuixingqingpitian’ | 2009-06-08 | 19.2±0.15 | 16.3±0.33 | 0.27±0.02 |
| ‘Xingquimili’ | 2009-06-03 | 23.3±0.36 | 17.6±0.16 | 0.24±0.03 |
| ‘Yamulong’ | 2009-06-08 | 33.1±0.27 | 18.0±0.18 | 0.34±0.03 |
| ‘Yongxing No.2’ | 2009-06-05 | 21.0±0.25 | 19.0±0.21 | 0.31±0.04 |
| ‘Feizixiao’ | 2009-06-10 | 26.2±0.30 | 17.5±0.22 | 0.44±0.03 |
| ‘Sanyuehong’ | 2009-05-14 | 25.4±0.27 | 14.9±0.17 | 0.35±0.02 |
| ‘Meiguili’ | 2009-06-13 | 19.9±0.27 | 20.2±0.17 | 0.31±0.03 |
| ‘Baila’ | 2009-05-29 | 21.8±0.26 | 17.1±0.23 | 0.18±0.01 |
| ‘Baitangying’ | 2009-05-29 | 23.3±0.29 | 16.7±0.26 | 0.15±0.00 |
| ‘Guiwei’ | 2009-06-18 | 21.7±0.28 | 17.0±0.26 | 0.27±0.02 |
| ‘Nuomici’ | 2009-06-27 | 25.7±0.31 | 17.1±0.23 | 0.30±0.01 |
| ‘Guinuo’ | 2009-07-04 | 23.3±0.36 | 16.1±0.23 | 0.36±0.03 |

Data are means of 30 fruits with the same sampling date of Table 1.
