# Supplementary material for: Differential Expression of Anthocyanin Biosynthetic Genes in Relation to Anthocyanin Accumulation in the Pericarp of Litchi Chinensis Sonn
Source: PLoS One. 2011 Apr 29;6(4):e19455. doi: 10.1371/journal.pone.0019455 (PMC3084873; doi:10.1371/journal.pone.0019455)
Supplement: Table S5 — Cloning of LcCHS, LcCHI, LcF3H, LcDFR, LcANS and LcUFGT. (DOC) [file pone.0019455.s006.doc]

Table S5 Cloning of *LcCHS*, *LcCHI*, *LcF3H*, *LcDFR*, *LcANS* and *LcUFGT*

S5-1 **Results of sequencing (Red letters represent binding domains with the primers)**

***LcCHS*** 5’-GAGAAGTTCAAGCGCATGTGTGACAAATCTATGATAAAGAAGCGTTACATGCATCTAACGGAAGATATTCTGAAGGAGAACCCTAACATGTGTGCATACATGGCGCCATCTATAGATGCCCGTCAAGACATTGTGGTGGTGGAGGTACCAAAGTTGGGGAAAGAAGCTGCACAGAAGGCGATCAAAGAGTGGGGCCAACCCAAATCCAAGATCACCCATCTCGTCTTCTGTACAACCTCAGGTGTAGACATGCCTGGCGCTGACTACCAGCTGACCAAGCTACTCGGTCTACGACCCTCAGTCAAGCGCCTCATGATGTACCAGCAGGGTTGCTTTGCTGGTGGGACAGTCCTCCGTCTCGCTAAAGACTTGGCGGAAAACAACAAGGGTGCACGTGTCCTTGTCGTCTGTTCCGAAATCACTGCAGTCACTTTCCGTGG-3***’***

***LcCHI***

5’-TTCCTCGGCGGCGCAGGGGAGAGAGGTTTGGAGATCCAAGGGAAATTCGTTAAGTTCACGGCGATCGGAGTTTACTTGGAGGATGTCGCTGTGTCGTCGCTCGCCGTTAAGTGGAAGGGCAAGACTGCCGAGGAGTTGACGGAATCCGTCGAGTTTTTCAGAGATATCGTTACTGGTCCCTTCGAGAAATTCATGAAGGTGACGATGATCTTGCCATTAACGGGTCAACAATACTCTGAGAAGGTCACTGAAAATTGCATCGCCTTTTGGAAATCCATTGGAATTTACACTGATGCAGAAG-3’

***LcF3H***

5’-TGGCGTGAAATAGTGACCTATTTCTCATACCCGATGAGGACCCGAGACTATTCAAGGTGGCCCGACAAGCCACAAGGGTGGATAGATGTGACAAAGGAGTACAGTGACAAGTTAATGGGGCTTGCATGCAAGCTTCTTGAGGTTTTATCAGAGGCAATGGGATTGGAGAAGGAGGCATTGACTAATGCATGCGTGGACATGGACCAAAAAGTTGTCGTCAATTACTATCCAAAATGCCCACAACCTGACCTCACTCTGGGACTCAAACGCCACACCGACCCGGGAACCATCACACTATTACTCCAGGATCAGGTTGGTGGGCTGCAGGCCACCAGAGACAATGGCAAGACTTGGATCACTGTTCAACCAGTTGATGGAGCCTTTGTGGTCAACCTTGGAGACCATGGCCATTATTTGAGCAATGGGAGGTTCAAGAA-3’

***LcDFR***

5’-GAGTCCAAAGATCCCGAAAACGAAGTAATAAAGCCAACTATCAATGGGATATTAGACATAATGAAGGCATGCAAGAAGGCAAAAACAGTGAAAAGGTTGGTGTTCACGTCATCAGCAGGAACTATGGATGTTGAAGAGCACAAAAAGCCTGTTTATGATGAGACTTGCTGGAGTGATATGGGCTTTGTCAGGTCCGTAAAGATGACTGGATGGATGTACTT-3’

***LcANS***

5’-AAGGAGAAGTATGCAAATGACCAGGCTTCAGGTAACGTTCAAGGCTATGGCAGCAAGCTAGCTAATAATGCTAGTGGCCAACTTGAGTGGGAGGACTATTTCTTCCATCTTATTTATCCTGAAGACAAGAGGGACTTGTCCATTTGGCCCAAGACACCTAAAGATTACATAGAGGTAACGAGCGAATATGCAAGGCAATTGAGAAGCTTGGCGACGAAGATCCTCTCAGTGCTATCACTAGGGTTAGGATTAGAAGAAGGAAGGCTAGAAAAGGAAGTTGGTGGTCTGGAAGAACTGCTGCTTCAGATGAAGATCAACTACTATCCAAAATGCCCACAACCAGAACTTGCTCTCGGTGTTGAAGCTCACACGGACGTCAGTGCACTCACTTTCATACTCCACAACATGGTTCCCGGGCTGCAACTCTTCTACGAAGGCAAGTGGATCACTGCCAAATGTGTTCCAAACTCCATCATCATGCACATTGGGGACACCATTGAAATCCTCAGCAACGGCAAGTACAAGAGCATTCTTCACAGGGGACTTGTCAACAAGGAAAAAGTGAGGATCTCGTGGGCTGTTTTCTGTGAACCACCAAAGGACAAGAT-3’

***LcUFGT***

5’-TCATGTGGCCGTCCTGGCCTTTCCATTCTCCACACATGCTGCTCCTCTCCTCTCCATTATCAGCCGCCTAGCCTCCTCTGCTCCAAACACTCATTTCTCATTCTTCAGCACTGCAGAATCCAACAACTCCCTCTTGTCTACACACAAACACTACTTCCTTCCCAATGTGAAAGCTTATAATGTTTCCAATGGGGTTCCGGATCACTATGTGTTCCTTGGGAAGCCTCAGGAGGACATTGAGTTGTTCATGAAGGCAGCTCCTGAGAACTTGAGGAAGGCAGTGGCGAAGGCTGCAGTGGAGACAAAGAGAAAGGTGAGTTGTTTGGTTACTGATAGTTTCCTTTGGTTCGCAGCAGAAATGGCAGAGGAGATGCAGGTGCCTTTCGTGCCTTGTTGGTTATCAGGGTCTAGCTCACTCTCGACTCATTTTTATACTGATGTTATCAGGGAAAAGATAGGACTTGAAGGAATTGAAGGAAGAGAAGATGAGCAGCTTAAATTCATTCAAGGAATGTCAAAAGTGTGCATTAGAGACTTGCCTGAAGGAGTCCTCTTTGGAAACTTGCAATCAGTATTTTCCGATATGCTACATCGAATGGGCCTGAAACTACCACGAGGGGACGCAGTTGTCATAAACAGCTTTGAAGAATTAGACCCTACAATAAACAATGATCTCAAATCCAAATTCAAACAGTTTCTCAACGTTGGTCCCTTCAATCTAATCTCTCCGCCACCAGCGGTTCCTAATACCAGCAGCTGCCTACCGTGGCTCGACAGGCAGAAGCCTGCATCCGTGGCATATCTTGGGTTCGGTTCTGTCTCAAGGCTGTCTCCTAATGAGATTGTAGCAGTAGCAGAGGCATTGGAAGCAAGTAAACTGTCATTTATATGGTCACTGAAGAAGAATCTACAAGCACATTTGCCAAATACAAAGTTGAATGGAATTGTGGTGGAATGGGCTCCTC-3’

**S5-2 PCR primers for cloning the 3’ end of full sequences**

| **Primer’s Name** | **Primer Sequences(5' to 3')** | **Product size(bp)** | **Anneal** [**Temperature**](http://www.iciba.com/temperature/)**（℃）** |
| --- | --- | --- | --- |
| *LcCHS*-3-F1 | gcgaagttccagctcatgtgtga | 1050 | 56 |
| *LcCHS*-3-F2 | CTCAGTCAAGCGCCTCATGATGT | 770 | 56 |
| *LcCHI*-3-F1 | TTCCTCGGCGGCGCAGGGGWGAG | 900 | 57 |
| *LcCHI*-3-F2 | CGGAGTTTACTTGGAGGATGT | 850 | 56 |
| *LcF3H-3*-F1 | AGATGTGACAAAGGAGTACAGTG | 830 | 57 |
| *LcF3H*-3-F2 | GATCACTGTTCAACCAGTTGAT | 560 | 55 |
| *LcDF*R-3-F1 | ATAAAGCCAACTATCAATGGGAT | 1000 | 57 |
| *LcDFR*-3-F2 | GTCATCAGCAGGAACTATGGAT | 900 | 56 |
| *LcANS*-3-F1 | AGGAAGTTGGTGGTCTGGAAG | 680 | 57 |
| *LcANS*-3-F2 | GCAACGGCAAGTACAAGAGCA | 440 | 56 |
| *LcUFGT*-3-F1 | GTTGGTCCCTTCAATCTTATC | 750 | 57 |
| *LcUFGT*-3-F2 | GTAGCAGAGGCATTGGAAGCA | 600 | 56 |

**S5-3 PCR primers for cloning the 5’ end of full sequences**

| **Primer’s Name** | **Primer Sequences(5' to 3')** | **Product size(bp)** | **Anneal** [**Temperature**](http://www.iciba.com/temperature/)**（℃）** |
| --- | --- | --- | --- |
| *LcCHS*-5-R1 | TGAATAGGGACACTGTGCAACA | 1150 | 56 |
| *LcCHS*-5-R2 | TGATGCCAATGGGAGTGAAT | 850 | 55 |
| *LcF3H*-5-R1 | GTCCTTGCTCATCTTCCTCCTGTA | 1000 | 56 |
| *LcF3H*-5-R2 | CAAGGTTGACCACAAAGGCTCCAT | 850 | 58 |
| *LcANS*-5-R1 | CCGTTGCTGAGGATTTCAATGGTG | 950 | 57 |
| *LcANS*-5-R2 | GCAGCAGTTCTTCCAGACCACCAA | 700 | 56 |
| *LcUFGT*-5-R1 | CTGCGTCTCCTTGTGGTAGTTG | 900 | 56 |
| *LcUFGT*-5-R2 | TGATAACCAACAAGGCACGAAAG | 500 | 56 |
