# Supplementary material for: Differential Expression of Anthocyanin Biosynthetic Genes in Relation to Anthocyanin Accumulation in the Pericarp of Litchi Chinensis Sonn
Source: PLoS One. 2011 Apr 29;6(4):e19455. doi: 10.1371/journal.pone.0019455 (PMC3084873; doi:10.1371/journal.pone.0019455)
Supplement: Table S6 — Cloning and identification of LcActin. (DOC) [file pone.0019455.s007.doc]

**Table S6 Cloning and identification of** *LcActin*

S 6-1Total RNA isolated from *Litchi* *chinensis* Sonn. cv. Nuomici


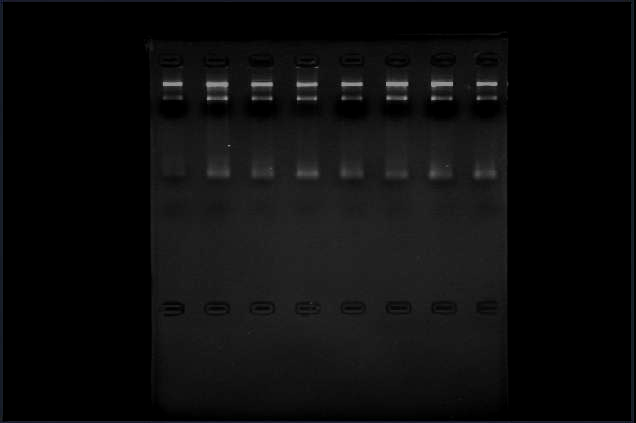


S6-2 Primers and Reference

Q*LcactinF* : ATGGCCGATGCTGAGGACATTC（5’→3’）

Q*LcactinR* : TCAGAAGCACTTCCTGTGGACAATG（5’→3’）

Full-Length：1134bp

**Reference：**Xu JT, Zhang Z, Peng RH, et al. Cloning and analysis of *MrACT* from *Malus micromalus*. Journal of Fruit Science, 2008, 25(3): 289-292

**S6-3 Electrophoresis** [**result**](http://www.iciba.com/result/) **of PCR segment**


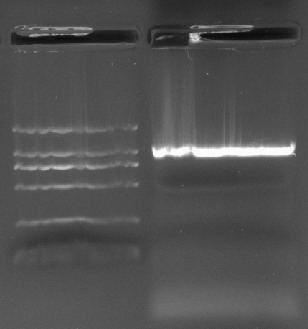


**2000bp**

**1000bp**

**750bp**

1 2

Lane 1：DL2000 Marker；Lane 2：PCR segment

S6-4 DNA sequencing (Beijing Genomics Institute for sequencing**)**

Result：(Full-Length 1134bp, Red letters represent binding domains with the primers)

atggccgatgctgaggacattcagcctctcgtttgcgacaatggaactgggatggtgaaggctggatttgctggggatgatgcccccagggcggtgtttcccagtattgttggtcggccccgacacactggtgtgatggttggtatgggtcagaaggatgcctatgttggtgatgaggcccaatcgaaaagaggtattcttaccttgaaatacccgattgaacatggtattgtcagcaactgggatgatatggaaaagatctggcatcacactttctacaatgagcttcgtgttgctccagaagagcacccggtgcttcttactgaggctcctttaaatcccaaggccaacagagagaagatgacccagattatgtttgaaacttttaatgtgcctgccatgtatgttgccatccaggccgtcctctctctttacgccagtggtcgcacaactggtatcgtgctggattctggtgatggtgtgtctcatactgtgccaatctatgaaggatatgcccttcctcatgctattctccgtttggacctggctggtcgtgatctcaccgatgcattgatgaagattctgaccgagagaggttacatgtttaccaccactgccgaacgggaaattgtccgtgacatgaaagagaagcttgcctatgtcgcgctagactatgagcaggaacttgagactgccaagagcagctcctctgtggagaagaactatgagttacctgatggtcaggtcatcactattggagctgagagattccgttgcccagaagtcctcttccagccatctctcatcggaatggaagctgctggtatccatgagactacctacaactccatcatgaagtgtgatgtggatatcaggaaggatctctatggtaacattgtgctcagtggtggttctactatgttccctggtattgccgaccgtatgagcaaggaaatcactgcccttgccccaagcagcatgaagatcaaggttgtggctccacctgagagaaaatacagtgtctggattggaggatcaatcctcgcatctctcagcaccttccaacagatgtggatttcaaaggatgagtacgacgagtctggtccatccattgtccacaggaagtgcttctga

S6-5 Alignment and phylogenetic analysis

| **Accessions** | **Description** | **[Max](http://blast.ncbi.nlm.nih.gov/Blast.cgi?CMD=Get&ALIGNMENTS=100&ALIGNMENT_VIEW=Pairwise&DATABASE_SORT=0&DESCRIPTIONS=100&FIRST_QUERY_NUM=0&FORMAT_OBJECT=Alignment&FORMAT_PAGE_TARGET=&FORMAT_TYPE=HTML&GET_SEQUENCE=yes&I_THRESH=&MASK_CHAR=2&MASK_COLOR=1&NEW_VIEW=yes&NUM_OVERVIEW=100&OLD_BLAST=false&PAGE=Translations&QUERY_INDEX=0&QUERY_NUMBER=0&RESULTS_PAGE_TARGET=&RID=E8XYN21R014&SHOW_LINKOUT=yes&SHOW_OVERVIEW=yes&STEP_NUMBER=&WORD_SIZE=3&DISPLAY_SORT=1&HSP_SORT=1#sort_mark)**  **[score](http://blast.ncbi.nlm.nih.gov/Blast.cgi?CMD=Get&ALIGNMENTS=100&ALIGNMENT_VIEW=Pairwise&DATABASE_SORT=0&DESCRIPTIONS=100&FIRST_QUERY_NUM=0&FORMAT_OBJECT=Alignment&FORMAT_PAGE_TARGET=&FORMAT_TYPE=HTML&GET_SEQUENCE=yes&I_THRESH=&MASK_CHAR=2&MASK_COLOR=1&NEW_VIEW=yes&NUM_OVERVIEW=100&OLD_BLAST=false&PAGE=Translations&QUERY_INDEX=0&QUERY_NUMBER=0&RESULTS_PAGE_TARGET=&RID=E8XYN21R014&SHOW_LINKOUT=yes&SHOW_OVERVIEW=yes&STEP_NUMBER=&WORD_SIZE=3&DISPLAY_SORT=1&HSP_SORT=1#sort_mark)** | **[Total score](http://blast.ncbi.nlm.nih.gov/Blast.cgi?CMD=Get&ALIGNMENTS=100&ALIGNMENT_VIEW=Pairwise&DATABASE_SORT=0&DESCRIPTIONS=100&FIRST_QUERY_NUM=0&FORMAT_OBJECT=Alignment&FORMAT_PAGE_TARGET=&FORMAT_TYPE=HTML&GET_SEQUENCE=yes&I_THRESH=&MASK_CHAR=2&MASK_COLOR=1&NEW_VIEW=yes&NUM_OVERVIEW=100&OLD_BLAST=false&PAGE=Translations&QUERY_INDEX=0&QUERY_NUMBER=0&RESULTS_PAGE_TARGET=&RID=E8XYN21R014&SHOW_LINKOUT=yes&SHOW_OVERVIEW=yes&STEP_NUMBER=&WORD_SIZE=3&DISPLAY_SORT=2&HSP_SORT=1#sort_mark)** | **[Query coverage](http://blast.ncbi.nlm.nih.gov/Blast.cgi?CMD=Get&ALIGNMENTS=100&ALIGNMENT_VIEW=Pairwise&DATABASE_SORT=0&DESCRIPTIONS=100&FIRST_QUERY_NUM=0&FORMAT_OBJECT=Alignment&FORMAT_PAGE_TARGET=&FORMAT_TYPE=HTML&GET_SEQUENCE=yes&I_THRESH=&MASK_CHAR=2&MASK_COLOR=1&NEW_VIEW=yes&NUM_OVERVIEW=100&OLD_BLAST=false&PAGE=Translations&QUERY_INDEX=0&QUERY_NUMBER=0&RESULTS_PAGE_TARGET=&RID=E8XYN21R014&SHOW_LINKOUT=yes&SHOW_OVERVIEW=yes&STEP_NUMBER=&WORD_SIZE=3&DISPLAY_SORT=4&HSP_SORT=0#sort_mark)** | **[E value](http://blast.ncbi.nlm.nih.gov/Blast.cgi?CMD=Get&ALIGNMENTS=100&ALIGNMENT_VIEW=Pairwise&DATABASE_SORT=0&DESCRIPTIONS=100&FIRST_QUERY_NUM=0&FORMAT_OBJECT=Alignment&FORMAT_PAGE_TARGET=&FORMAT_TYPE=HTML&GET_SEQUENCE=yes&I_THRESH=&MASK_CHAR=2&MASK_COLOR=1&NEW_VIEW=yes&NUM_OVERVIEW=100&OLD_BLAST=false&PAGE=Translations&QUERY_INDEX=0&QUERY_NUMBER=0&RESULTS_PAGE_TARGET=&RID=E8XYN21R014&SHOW_LINKOUT=yes&SHOW_OVERVIEW=yes&STEP_NUMBER=&WORD_SIZE=3&DISPLAY_SORT=0&HSP_SORT=0#sort_mark)** | **[Max ident](http://blast.ncbi.nlm.nih.gov/Blast.cgi?CMD=Get&ALIGNMENTS=100&ALIGNMENT_VIEW=Pairwise&DATABASE_SORT=0&DESCRIPTIONS=100&FIRST_QUERY_NUM=0&FORMAT_OBJECT=Alignment&FORMAT_PAGE_TARGET=&FORMAT_TYPE=HTML&GET_SEQUENCE=yes&I_THRESH=&MASK_CHAR=2&MASK_COLOR=1&NEW_VIEW=yes&NUM_OVERVIEW=100&OLD_BLAST=false&PAGE=Translations&QUERY_INDEX=0&QUERY_NUMBER=0&RESULTS_PAGE_TARGET=&RID=E8XYN21R014&SHOW_LINKOUT=yes&SHOW_OVERVIEW=yes&STEP_NUMBER=&WORD_SIZE=3&DISPLAY_SORT=3&HSP_SORT=3#sort_mark)** |
| --- | --- | --- | --- | --- | --- | --- |
| [AAP73457.1](http://www.ncbi.nlm.nih.gov/protein/32186906?report=genbank&log$=prottop&blast_rank=3&RID=E8XYN21R014) | actin [*Gossypium hirsutum*] | [754](http://blast.ncbi.nlm.nih.gov/Blast.cgi#32186906%2332186906) | 754 | 99% | 0.0 | 99% |
| [ABR45727.1](http://www.ncbi.nlm.nih.gov/protein/149938964?report=genbank&log$=prottop&blast_rank=5&RID=E8XYN21R014) | ACT1 [*Actinidia deliciosa*] | [753](http://blast.ncbi.nlm.nih.gov/Blast.cgi#149938964%23149938964) | 753 | 99% | 0.0 | 99% |
| [ADA70361.1](http://www.ncbi.nlm.nih.gov/protein/281485191?report=genbank&log$=prottop&blast_rank=21&RID=E8XYN21R014) | actin [*Persea americana*] | [749](http://blast.ncbi.nlm.nih.gov/Blast.cgi#281485191%23281485191) | 749 | 99% | 0.0 | 98% |
| AAA80356.1 | actin-2 [*Arabidopsis thaliana*] | [749](http://blast.ncbi.nlm.nih.gov/Blast.cgi#15242516%2315242516) | 749 | 99% | 0.0 | 98% |
| [ACN54541.1](http://www.ncbi.nlm.nih.gov/protein/224552368?report=genbank&log$=prottop&blast_rank=23&RID=E8XYN21R014) | actin [*Carica papaya*] | [748](http://blast.ncbi.nlm.nih.gov/Blast.cgi#224552368%23224552368) | 748 | 99% | 0.0 | 98% |
| [ACJ04738.1](http://www.ncbi.nlm.nih.gov/protein/209979578?report=genbank&log$=prottop&blast_rank=41&RID=E8XYN21R014) | actin [*Sedum alfredii*] | [743](http://blast.ncbi.nlm.nih.gov/Blast.cgi#209979578%23209979578) | 743 | 99% | 0.0 | 97% |
| [NP_001054419.1](http://www.ncbi.nlm.nih.gov/protein/115461639?report=genbank&log$=prottop&blast_rank=43&RID=E8XYN21R014) | Os05g0106600 [*Oryza sativa* Japonica Group] | [743](http://blast.ncbi.nlm.nih.gov/Blast.cgi#115461639%23115461639) | 743 | 99% | 0.0 | 97% |
| AAW78915.1 | actin [*Triticum aestivum*] | [741](http://blast.ncbi.nlm.nih.gov/Blast.cgi#24496452%2324496452) | 741 | 99% | 0.0 | 96% |
| [BAD27408.1](http://www.ncbi.nlm.nih.gov/protein/50058115?report=genbank&log$=prottop&blast_rank=56&RID=E8XYN21R014) | actin [*Nicotiana tabacum*] | [741](http://blast.ncbi.nlm.nih.gov/Blast.cgi#50058115%2350058115) | 741 | 99% | 0.0 | 97% |

This gene sequence has been submitted to the NCBI GenBank and the accession number is HQ615689.
