# Supplementary material for: Differential Expression of Anthocyanin Biosynthetic Genes in Relation to Anthocyanin Accumulation in the Pericarp of Litchi Chinensis Sonn
Source: PLoS One. 2011 Apr 29;6(4):e19455. doi: 10.1371/journal.pone.0019455 (PMC3084873; doi:10.1371/journal.pone.0019455)
Supplement: Table S7 — Evaluating the expression stability of reference genes. (DOC) [file pone.0019455.s008.doc]

Table S7 Evaluating the expression stability of reference genes

S7-1 Primers of reference genes

| **Gene** | **Gene description** | **Forward primer (5' to 3')** | **Reverse primer (5' to 3')** |
| --- | --- | --- | --- |
| ***ACTIN*** | β-actin | GTGGTTCTACTATGTTCCCTG | CTCGTCGTACTCATCCTTTG |
| ***18S*** | 18S rRNA | ATTACCCAATCCTGACACGG | AACCCAAAGTCCAACTACGAG |
| ***GAPDH*** | Glyceraldehyde-3-phosphate dehydrogenase | GACAGCAGGTCAAGTATCT | GAAGCAGCCAAGCGACTT |
| ***25S*** | 25S rRNA | ATAACCGCATCAGGTCTCCAAG | CCTCAGAGCCAATCCTTTTCC |
| ***UBQ*** | Polyubiquitin | AAAGCTCCGACACCATTGAC | ATCCTCAAGCTGCTTTCCAG |
| ***eEF*** | Elongation factor | TTTCACTCTTGGTGTGAAGCAGAT | GACTTCCTTCACGATTTCATCGTAA |

S7-2 C_T_ MEAN of selected reference genes under all treatment conditions

|  | **Treatments** | **Days after treatment** | C_T_ MEAN of selected reference genes | | | | | |
| --- | --- | --- | --- | --- | --- | --- | --- | --- |
|  |  |  | ***ACTIN*** | ***18S*** | ***GAPDH*** | ***25S*** | ***UBQ*** | ***eEF*** |
| **S1** | CK | 7 | 21.694 | 11.855 | 21.409 | 12.875 | 21.843 | 34.955 |
| **S2** | ABA | 7 | 20.881 | 10.614 | 20.750 | 12.141 | 21.747 | 33.955 |
| **S3** | CPPU | 7 | 20.738 | 10.834 | 20.821 | 12.181 | 21.653 | 33.563 |
| **S4** | CK | 14 | 21.521 | 10.456 | 21.127 | 12.978 | 20.991 | 33.551 |
| **S5** | ABA | 14 | 20.770 | 11.017 | 22.068 | 13.337 | 20.835 | 32.639 |
| **S6** | CPPU | 14 | 21.321 | 11.838 | 23.034 | 12.459 | 21.918 | 33.634 |
| **S7** | CK | 21 | 20.752 | 12.144 | 22.912 | 12.119 | 21.287 | 32.507 |
| **S8** | ABA | 21 | 21.073 | 11.148 | 23.248 | 11.373 | 20.366 | 32.193 |
| **S9** | CPPU | 21 | 21.160 | 11.377 | 23.298 | 10.310 | 20.742 | 31.677 |
| **S10** | CK | 28 | 21.228 | 15.882 | 22.958 | 18.361 | 20.772 | 32.044 |
| **S11** | ABA | 28 | 21.180 | 11.361 | 23.405 | 11.590 | 21.069 | 32.516 |
| **S12** | CPPU | 28 | 21.936 | 10.810 | 23.894 | 12.668 | 21.566 | 33.157 |

S7-3 The expression stability values of selected reference genes calculated by the Normfinder software

| Gene name | Stability value |
| --- | --- |
| *25S* | 0.124 |
| *UBQ* | 0.038 |
| *eEF* | 0.044 |
| *18S* | 0.092 |
| *ACTIN* | 0.024 |
| *GAPDH* | 0.055 |
| Best gene | Actin |
